# Arterial spin labelling perfusion MRI analysis for the Human Connectome Project Lifespan Ageing and Development studies

**DOI:** 10.1162/imag_a_00444

**Published:** 2025-01-27

**Authors:** Thomas F. Kirk, Flora A. Kennedy McConnell, Jack Toner, Martin S. Craig, Davide Carone, Xiufeng Li, Yuriko Suzuki, Timothy S. Coalson, Michael P. Harms, Matthew F. Glasser, Michael A. Chappell

**Affiliations:** Sir Peter Mansfield Imaging Centre, University of Nottingham, Nottingham, United Kingdom; Quantified Imaging, London, United Kingdom; Radcliffe Department of Medicine, University of Oxford, Oxford, United Kingdom; Centre for Magnetic Resonance Research, University of Minnesota, Minneapolis, MN, United States; Wellcome Centre for Integrative Neuroimaging, FMRIB, Nuffield Department of Clinical Neurosciences, University of Oxford, Oxford, United Kingdom; Departments of Radiology and Neuroscience, Washington University School of Medicine, St. Louis, MO, United States; Department of Psychiatry, Washington University School of Medicine, St. Louis, MO, United States

**Keywords:** perfusion, arterial spin labelling (ASL), cerebral blood flow (CBF), arterial transit time (ATT), human connectome project (HCP), ageing

## Abstract

The Human Connectome Project Lifespan studies cover the development (5–21) and ageing (36–100+) phases of life. Arterial spin labelling (ASL) was included in the imaging protocol, resulting in one of the largest datasets collected to date of high spatial resolution multiple delay ASL covering 3,000 subjects. The human connectome project (HCP)-ASL minimal processing pipeline was developed specifically for this dataset to pre-process the image data and produce perfusion estimates in both volumetric and surface template space, though quality control is not performed. Applied to the whole dataset, the outputs of the pipeline revealed significant and expected differences in perfusion between the development and ageing cohorts. Visual inspection of the group average surface maps showed that cortical perfusion often followed cortical areal boundaries, suggesting differential regulation of cerebral perfusion within brain areas at rest. Group average maps of arterial transit time also showed differential transit times in core and watershed areas of the cerebral cortex, which are useful for interpreting haemodynamics of functional MRI images. The pre-processed dataset will provide a valuable resource for understanding haemodynamics across the human lifespan.

## 1 Introduction

The Human Connectome Project (HCP) Lifespan studies extend the original HCP (collected in young adults) to characterise brain organisation and connectivity during the development (HCD, 5–21 years) (Somerville et al., 2018) and ageing (HCA, 36–100+ years) (Bookheimer et al., 2019) phases of life. Taken together, the studies acquired comprehensive neuroimaging datasets for more than 3,200 individuals to facilitate characterisation of the organisation and connectivity of the brain at various life stages, including childhood, puberty, middle age, menopause, later life and the “oldest old”. The Lifespan studies included multiple delay arterial spin labelling (ASL) MRI as an imaging modality, which permits, for the first time, perfusion measurements to be included in the HCP data releases.

ASL is a non-invasive imaging technique that uses magnetically labelled blood as an endogenous tracer; when acquired at multiple delays (as was the case here), it permits measurement of both cerebral perfusion (CBF) and arterial transit time (ATT). Cerebral perfusion is known to vary substantially during early life (Avants et al., 2015;Satterthwaite et al., 2014), to decline with age (Kiely et al., 2022;Leidhin et al., 2021), and to be affected by many of the diseases common in older age (Alosco et al., 2013;Binnewijzend et al., 2016;Hu et al., 2010;Melzer et al., 2011;Ortapamuk & Naldoken, 2006;Patel & Markus, 2011;Prohovnik et al., 2007;Shi et al., 2016). Though ASL has been acquired in previous population studies such as the UK Biobank (Miller et al., 2016), TILDA (Donoghue et al., 2018), GENFI (Mutsaerts et al., 2019), PREVENT (Mak et al., 2021), and Whitehall II Imaging Sub-study (Suri et al., 2019), the HCP Lifespan ASL dataset is unique in its size and quality, in particular due to the use of an unusually high spatial resolution multiple delay acquisition.

When the HCP was launched in 2010, the approach adopted was novel: to acquire a large and high-quality dataset, to maintain that quality during minimal pre-processing, and then release the outputs for others to analyse further. Many aspects of this “HCP-Style” to acquisition and analysis have been adopted by other studies (Glasser, Smith, et al., 2016); importantly, it has led to improvements in the reproducibility and transparency of research because all parties can access the underlying data (Elam et al., 2021). The objective of the HCP-ASL pipeline is to provide similarly high-quality pre-processed perfusion measures that others may use for downstream analysis, and many of the design decisions have been made with equivalence to existing HCP pipelines in mind in the hope of exploiting the data quality to its full extent, particularly with regard to cross-subject alignment of cortical areas.

A notable feature of existing HCP pipelines is the use of surface-based analysis to improve the alignment of cortical areas across individuals and reduce partial volume effects in group analyses (Coalson et al., 2018;Glasser et al., 2013;Glasser, Smith, et al., 2016), though exploiting the advantages of this paradigm requires accurate segmentations of the cerebral cortex. For the HCP studies, this was achieved via the use of high-resolution T1- and T2-weighted anatomical scans at 0.8 mm isotropic resolution or better, which may not be feasible for all studies (Glasser, Smith, et al., 2016). Conversely, the volumetric approach to group analysis relies on volumetric registration which is known to be inaccurate at aligning cortical areas across most of the cortical sheet and substantially worsens partial volume effects across tissues in group analyses (Coalson et al., 2018). In keeping with the existing HCP pipelines, the HCP-ASL pipeline produces perfusion estimates in a surface-based representation so that surface techniques may be used in downstream analysis. As few studies have yet explored surface representation of ASL-derived measures (examples include (Taso et al., 2021;Verclytte et al., 2015)), the HCP-ASL pipeline will enable the community to examine the utility of such an approach, in combination with other functional data also on the surface. The pipeline also produces volumetric outputs for compatibility with conventional analysis workflows.

This work details the processing steps performed by the HCP-ASL minimal processing pipeline and presents summary perfusion statistics calculated across the HCP Lifespan cohorts. There are numerous strategies that may be used for processing ASL data, particularly multi-delay data, and there remains debate as to which approach is best (Alsop et al., 2015;Fan et al., 2024;Lindner et al., 2023;Woods et al., 2024). We have not tried to systematically resolve these differences in this work, and instead present the pipeline as a best effort for the unique nature of the dataset. For an example analysis of perfusion measurements derived from the data, the reader is referred toJuttukonda et al. (2021).

## 2 Materials and Methods

The HCP Lifespan ASL data were processed with the HCP-ASL pipeline version 0.1.2, available athttps://github.com/physimals/hcp-asl/releases/tag/v0.1.2. The following sections detail the data acquisition and the processing steps performed by the pipeline.

### 2.1 Data acquisition

ASL data for the HCP Lifespan studies was acquired in 5.5 min of scanning on five 3T Siemens Prisma scanners at four sites (Harms et al., 2018). Data were collected for 1,306 subjects for HCD (655 female, ages 5–21 years) and 1,199 for HCA (681 female, ages 36–102 years); the distribution across sites is given in thesupplementary material. Ethics approvals for the data collection were granted by the Washington University in St. Louis Institutional Review Board (HCA IRB ID #: 201603117, HCD IRB ID#: 201603135) and informed consent was obtained from all participants for being included in the study.

Pseudo-continuous ASL (PCASL) was used, with a 1.5 s label duration and multiple post-labelling delays (PLDs) of 0.2, 0.7, 1.2, 1.7, and 2.2 s, repeated 6, 6, 6, 10, and 15 times, respectively. A simultaneous multi-slice (SMS) 2D EPI acquisition with a multi-band factor of 6 was used without background suppression to achieve 2.5 mm isotropic resolution (2.27 mm slice thickness plus 10% gap), with 60 slices total, 59 ms readout time per slice, and 19 ms echo time (further details are given inLi et al. (2015)). Although 2D readouts have inherently lower signal-to-noise ratio than 3D readouts, they do not introduce substantial spatial blurring due to T_2_decay during the readout (Alsop et al., 2015;Woods et al., 2024). Such blurring would reduce the accuracy of registration between the ASL data and T1w anatomical image, negatively affecting the surface parameter maps that are the desired endpoint of the pipeline. Blurring can be reduced via the use of segmented 3D acquisitions, but such acquisitions are highly sensitive to motion. For this reason, after comparing 3D and 2D (with SMS) PCASL, HCP Lifespan adopted the latter (Harms et al., 2018).

To calibrate perfusion measurements into units of ml/100 g/min, two 2.5 mm isotropic PD-weighted M0 calibration images (TR > 8 s) were acquired at the end of the PCASL scan. In addition, a strong pre-saturation covering the imaging region was played out just before the PCASL module using three selective RF pulses with the spoiling gradients for each applied along two different directions (i.e., the first pre-saturation pulse was followed with spoiling gradients along the readout and slice directions; the second along the phase and slice directions; and the third along the readout and phase directions). For susceptibility distortion correction, two phase-encoding-reversed spin-echo images were acquired that were geometrically and distortion matched to the ASL data. T1- and T2-weighted anatomical and functional MRI images were acquired and pre-processed via the existing HCP minimal processing pipelines (Glasser et al., 2013,^2018^,^2019^;Harms et al., 2018;Robinson et al., 2018).

### 2.2 Data pre-processing and corrections

The ASL images contained several geometric and intensity artefacts that required correction before perfusion estimation could be performed. The use of a pre-saturation pulse in a multi-slice acquisition scheme contributed to slice-wise intensity variations in the inferior-superior direction in the ASL timeseries and calibration images, most notably between the last and first slices of adjacent bands, illustrated inFigure 1.

**Fig. 1.**
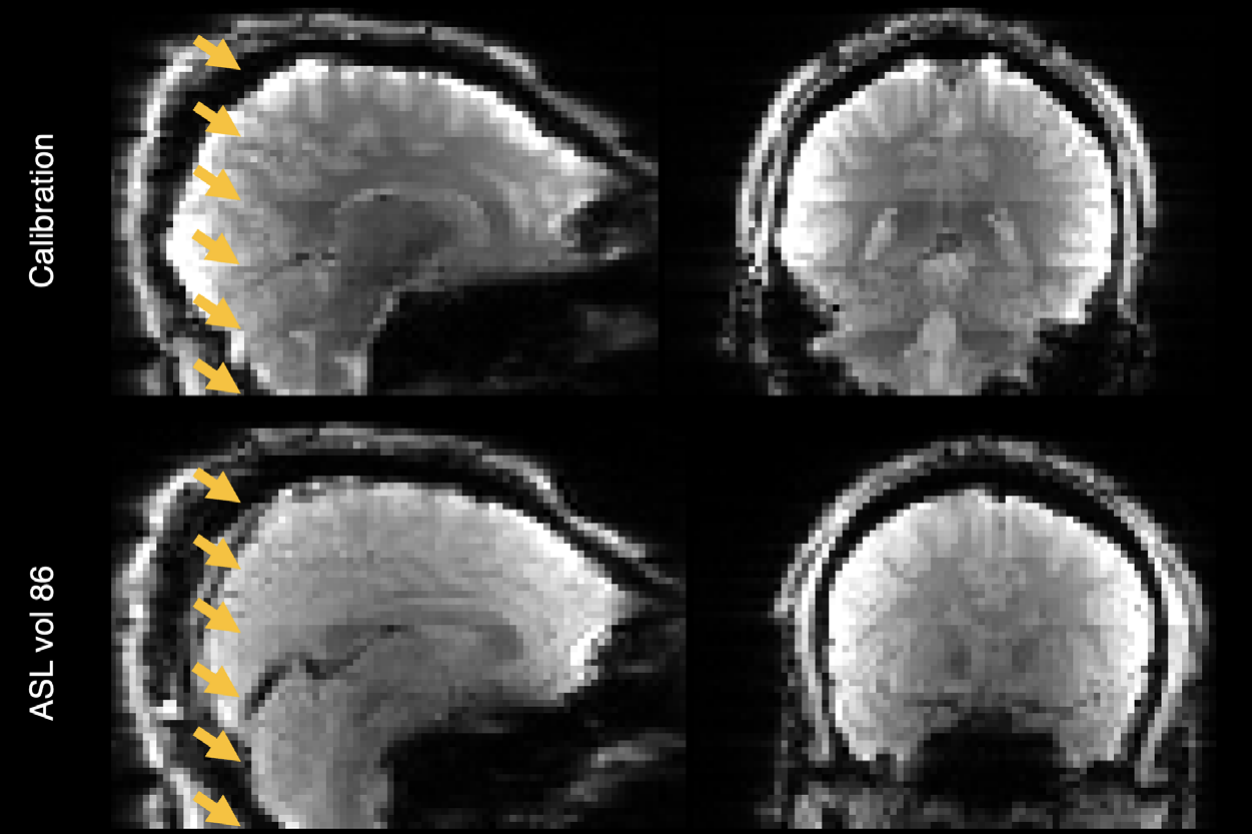
Banding artefact visible on the calibration image (top row) and last volume of the ASL timeseries (bottom row). The bottom of each band (six in total) is shown with an arrowhead.

The perfusion signal in ASL is obtained by subtracting successive pairs of label and control images in the ASL timeseries. This means that ASL is sensitive to within-timeseries motion which can lead to spurious signal when mis-aligned images are subtracted, and it is, therefore, necessary to motion correct the data. Conventional methods for rigid-body motion correction such as FSL MCFLIRT (Jenkinson et al., 2002) may not be accurate when applied to banded data because the registration cost function may reward alignment of the intensity banding rather than anatomy, leading to anatomical misalignment, which will produce spurious signal after label–control subtraction. Furthermore, within a single label–control pair that have relative motion, even if it were possible to perfectly re-align anatomy there would remain artefactual intensity variations due to the bands occurring at different anatomical locations across the pair, resulting in imperfect elimination of the banding effects after subtraction. It was, therefore, decided to “de-band” the data before performing motion correction.

The first banding correction applied to the data accounted for the different relaxation times of each slice in the 2D readout, which gives rise to a saturation recovery effect in the signal. While this effect exists in a conventional non-SMS 2D ASL acquisition (Golay et al., 2004), with SMS acquisitions there are adjacent slices within brain tissue acquired in the first versus last SMS excitation, making the discontinuity more evident, and importantly, contributing to the prominent banding artefact that was observed. To remove the saturation recovery effect, a simplified model of the form$S\left(t\right)\;=\;1\;-\;e^{-\frac{t}{T_{1}}}$was fitted to the control images of the ASL timeseries using FSL FABBER, where*T_1_*is the longitudinal relaxation time of tissue and*t*the slice-specific interval from the pre-saturation module (which is the sum of the duration of the labelling module, the PLD, and the slice-specific delay in the readout module, accounting properly for the SMS nature of the acquisition). An example T1 map generated by FABBER is given in thesupplementary material. The saturation recovery differences were then corrected (in both the control and label images) by normalising the intensities to the estimated value that they would have had if all the slices had been acquired simultaneously at the PLD.

During pipeline development, it was found that saturation recovery correction alone was unable to fully remove banding in both the calibration image and later PLDs of the ASL timeseries (illustrated in the Results section). Given the long TR of the calibration image (8 s), or long recovery times of the later PLDs (up to 3.7 s), theory predicts that longitudinal magnetisation will have largely recovered following those intervals and, therefore, the saturation recovery effect will be minimal. The presence of banding in the calibration images and later PLDs implied that there was a second banding mechanism (possibly a magnetisation transfer effect, addressed in the Discussion section), for which a correction was empirically derived using the calibration images of 80 subjects drawn equally from the HCA and HCD cohorts. After performing saturation recovery correction and masking the images to include grey and white matter only, a linear model for slice-wise mean intensity was fitted to each band and the coefficients averaged across subjects (seeSupplementary Materialfor details).

Correction for gradient non-linearity distortion was performed using*gradunwarp*operating on the calibration image with the gradient coefficients file for the Prisma system (as used in the HCP*PreFreeSurfer*pipeline (Glasser et al., 2013)). Susceptibility distortion correction was performed using FSL TOPUP operating on a pair of phase-encode reversed spin-echo images that had undergone gradient distortion correction. Bias field (B1) estimation was performed using the spin echo-based approach (“SEBASED”,Glasser et al., 2016) operating on the calibration image that had undergone gradient distortion, susceptibility distortion, and banding correction.

**Fig. 2.**
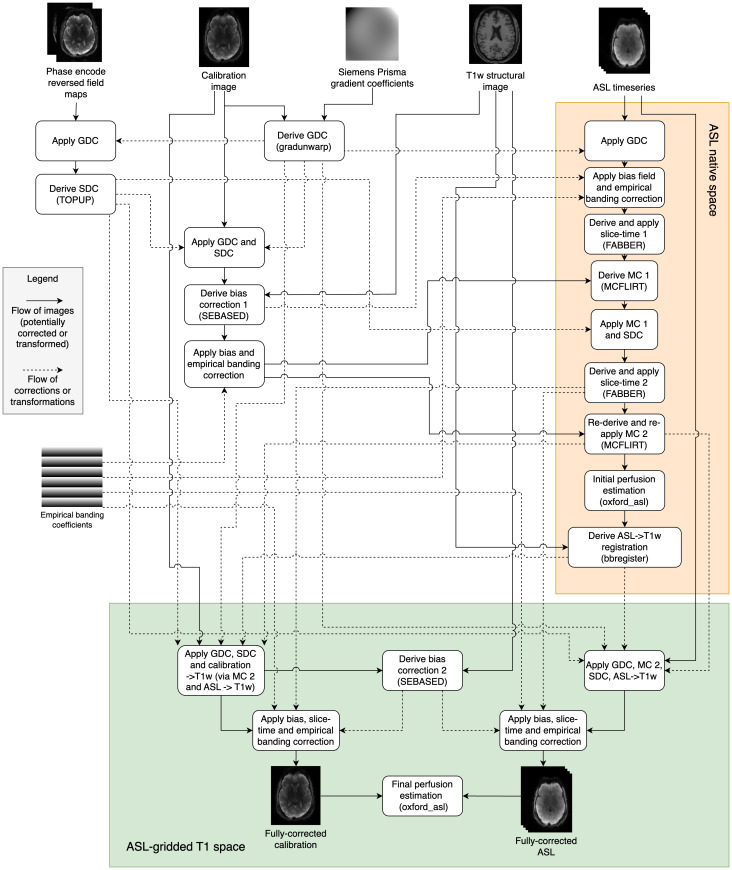
Schematic diagram showing how the fully corrected calibration and ASL images in ASL-gridded T1 space are derived (green box). Note that some intermediate registrations and transformations are not shown for clarity (particularly in the orange shaded box); all operations applied to the fully corrected outputs are shown. SEBASED: spin-echo based, SDC: susceptibility distortion correction, GDC: gradient distortion correction, MC: motion correction, FABBER: FSL model-fitting tool, MCFLIRT: FSL motion correction tool.

Figure 2shows how the aforementioned preprocessing corrections were derived and combined in the HCP-ASL pipeline to produce the fully corrected ASL and the calibration images on which perfusion estimation can be performed. Though two calibration images were acquired, only the first was used in the pipeline, though both were fully corrected. Some corrections were refined iteratively: for example, given that banding is expected to have a strong influence on the accuracy of motion estimation, each was performed twice on the assumption that an improvement in one should lead to an improvement in the other. Motion estimation was performed using MCFLIRT with the calibration image as the reference to yield a registration between the calibration image and ASL timeseries. In total, four subject-specific inputs and two generic inputs to the pipeline are required: for the individual, the phase encode reversed field maps, the calibration image, the T1w structural image, and the ASL timeseries, and generically, the Siemens Prisma gradient coefficients and the empirical banding correction coefficients.

### 2.3 Data alignment within individuals

In keeping with HCP fMRI pipelines, the HCP-ASL pipeline transforms ASL timeseries data into alignment with the structural images before analysis (Glasser et al., 2013), though retains the original 2.5 mm resolution (hereafter referred to as “ASL-gridded T1 space”). Multiple steps were used to obtain an accurate ASL to T1w registration. An initial registration was made using FreeSurfer’s boundary-based registration (BBR,*bbregister*) between the calibration image and the T1w image (Greve & Fischl, 2009;Jenkinson et al., 2002); this was combined with the motion correction matrices to obtain a registration between ASL and T1w. The calibration image has better grey–white tissue contrast than the un-subtracted ASL timeseries which facilitates accurate BBR. Then, perfusion estimation on the native ASL data with all banding and distortion corrections was performed to obtain a CBF map with increased grey–white tissue contrast, on which a second BBR was performed to the T1w image. The CBF map from this step was then discarded.

The transform between the ASL and calibration images and T1w alignment was merged with the motion correction, susceptibility distortion, and gradient distortion transforms and applied in a single step to the raw ASL and calibration images using the*regtricks*library to produce the fully transformed ASL and calibration images in ASL-gridded T1 space (Kirk, 2022). Jacobian intensity correction for distortion-induced signal pile-up was performed and cubic splines with pre-filtering were used to perform the interpolation (Unser et al., 1993). Minimising the number of interpolation operations aimed to reduce the introduction of additional partial volume effects that cannot be entirely removed with subsequent correction. SEABASED bias field estimation was re-run on the fully transformed calibration image to obtain bias field correction coefficients in ASL-gridded T1 space.

To intensity correct (banding and bias) the fully transformed ASL and calibration images, the calibration to T1w registration was applied to the empirical banding coefficients, and the ASL to T1w registration to the saturation recovery correction coefficients, to transform both banding corrections into ASL-gridded T1 space. Given the ASL and calibration image data were distortion corrected, no distortion correction was applied to the banding correction coefficients (it is only necessary to correct one or the other). The empirical banding coefficients were combined with the newly derived bias field estimates to intensity correct the calibration image, and both sets of coefficients were combined with the bias field estimates to intensity correct the ASL image.

### 2.4 Perfusion analysis

In the presence of head motion, voxels travelling between neighbouring slices during the acquisition will receive differing intensity scaling during the banding corrections, which could lead to spurious signal after label–control subtraction. The general linear model (GLM) framework for motion-aware subtraction of banded and background-suppressed ASL data developed bySuzuki et al. (2019)was used in this work, with the small modification that it was not necessary to account for background suppression because it was not used during the image acquisition. The subtracted timeseries data were then used for perfusion estimation.

CBF and ATT estimation were performed using a variational Bayesian method via the*oxford_asl*script (Chappell et al., 2009,^2023^). The*aslrest*Buxton model with CBF, ATT, and macrovascular components was used (Buxton et al., 1998;Chappell et al., 2011). A normal distribution prior with mean 1.3 s was used on ATT and an automatic relevancy determination (ARD) prior was used on macrovascular blood volume to remove this component from non-arterial voxels. Slice-timing correction was performed by adjusting the PLDs in each voxel by their slice-timing offset, and perfusion was converted from arbitrary units into ml/100 g/min using the mean signal value of CSF in the lateral ventricles from the calibration image (Pinto et al., 2020). Reference region calibration was used instead of a voxel-wise strategy due to the availability of a high-resolution calibration image, which allowed for accurate ventricular segmentation with minimal PVE, as well as mitigating the possibility of introducing (potentially uncorrected) banding artefacts from the calibration image if voxel-wise division were used. The tissue T1 values previously estimated for saturation recovery correction were passed to*oxford_asl.*

ASL is typically acquired with ~4 mm voxel sizes which gives rise to partial volume effects (PVE) caused by the coarse spatial resolution of the data in relation to the cerebral cortex, which has a mean thickness of around 2.5 mm. There is ongoing debate as to whether PVE correction (PVEc) should be routinely used in ASL studies. Though it is theoretically justified, particularly for studies covering a wide range of participant ages, there remain questions around the robustness and efficacy of currently available methods (Chappell et al., 2021). The ASL data acquired by the Lifespan HCP had a relatively high spatial resolution (2.5 mm isotropic), which not only reduced the PVE but also enabled more accurate PVEc to be performed. PVEc is performed in the voxel space using a spatial variational Bayes method implemented in*oxford_asl*(Chappell et al., 2011); the required partial volume estimates are obtained using Toblerone operating on the FreeSurfer-derived cortical surfaces and subcortical segmentations (Fischl, 2012;Kirk et al., 2020). For PVEc, a normal distribution prior with mean of 1.3 s was used for GM ATT and 1.6 s for WM ATT.

**Fig. 3.**
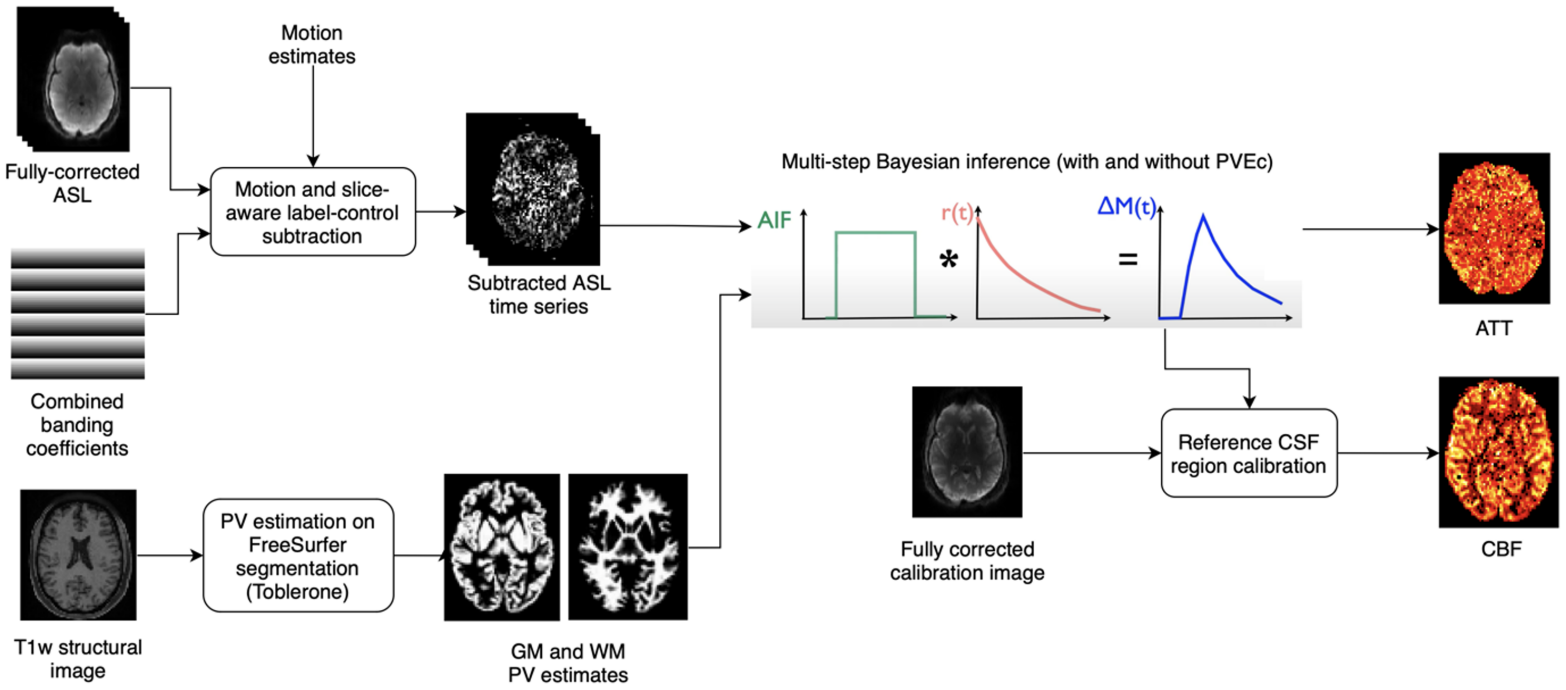
Perfusion analysis applied to ASL difference data after alignment with the individual’s T1w image. Bayesian inference is used to produce both partial volume-corrected and non-corrected estimates of perfusion and arterial arrival times, as well as arterial cerebral blood volume estimates. During inference, the slice-timing image on the right is used to produce slice-specific PLDs. Calibration of perfusion measures was performed using CSF reference region calibration.

Both PVEc and non-PVEc perfusion estimates are generated so that the end user may decide which is most appropriate for their application. For non-PVEc, spatial regularisation is not used (deviating from the recommended settings for*oxford_asl*, which were otherwise used) to minimise the mixing of signal across the grey/white cortical boundary, at the cost of somewhat greater image noise. By contrast, for PVEc, spatial regularisation is an intrinsic feature of the spatial variational Bayes method used by*oxford_asl*.

### 2.5 Output in standard space; calculation of image-derived phenotypes

Volumetric CBF and ATT maps from*oxford_asl*are produced in both ASL-gridded T1w space and MNI152 2 mm template space, via the existing FNIRT registration produced by the HCP PreFreeSurfer structural processing pipeline. To produce output parameter maps in greyordinates space (a combined surface and volumetric space represented in CIFTI format), the volumetric outputs of*oxford_asl*are projected onto the individual’s native cortical surface using the HCP ribbon-constrained method, registered with MSMAll multi-modal areal-feature-based registration and resampled to a common surface mesh, and finally smoothed with a 2 mm full-width half-maximum kernel using a surface-constrained method (Glasser et al., 2013,^2016^;Robinson et al., 2014,^2018^). The surface and MNI outputs (masked to consider subcortical structures only) were combined to produce the final CIFTI greyordinates files. Image-derived phenotypes (IDPs) were produced for each participant by computing the mean and standard deviation within each parcel of the HCP multi-modal parcellation (version 1.0) for both PVEc and non-PVEc variants of CBF and ATT.

### 2.6 Quality control

Though the HCP-ASL pipeline does not perform any automated QC, it does produce intermediate outputs that enable researchers to perform visual checks. These are produced as a single Workbench scene file (a pre-specified visualisation) containing seven scenes, each of which is saved as a standalone image to facilitate quick inspection (although full exploration requires opening the scene in Workbench). The scenes illustrate (a) the bounding box of the ASL timeseries after motion correction within the acquisition field of view (voxels missing data due to their movement outside of the acquisition field-of-view for a single timepoint are excluded from perfusion estimation) and the quality of registration to the T1w scan, illustrated inFigure 4; (b) the raw ASL timeseries before and after all corrections are applied; (c) non-PVEc CBF displayed on the inflated cortical surface and in subcortical structures; (d) non-PVEc ATT displayed in the same manner; (e) PV estimates for GM and WM, and the ventricular CSF mask used for calibration; (f) a volumetric montage of non-PVEc CBF; and (g) a volumetric montage of non-PVEc ATT.

**Fig. 4.**
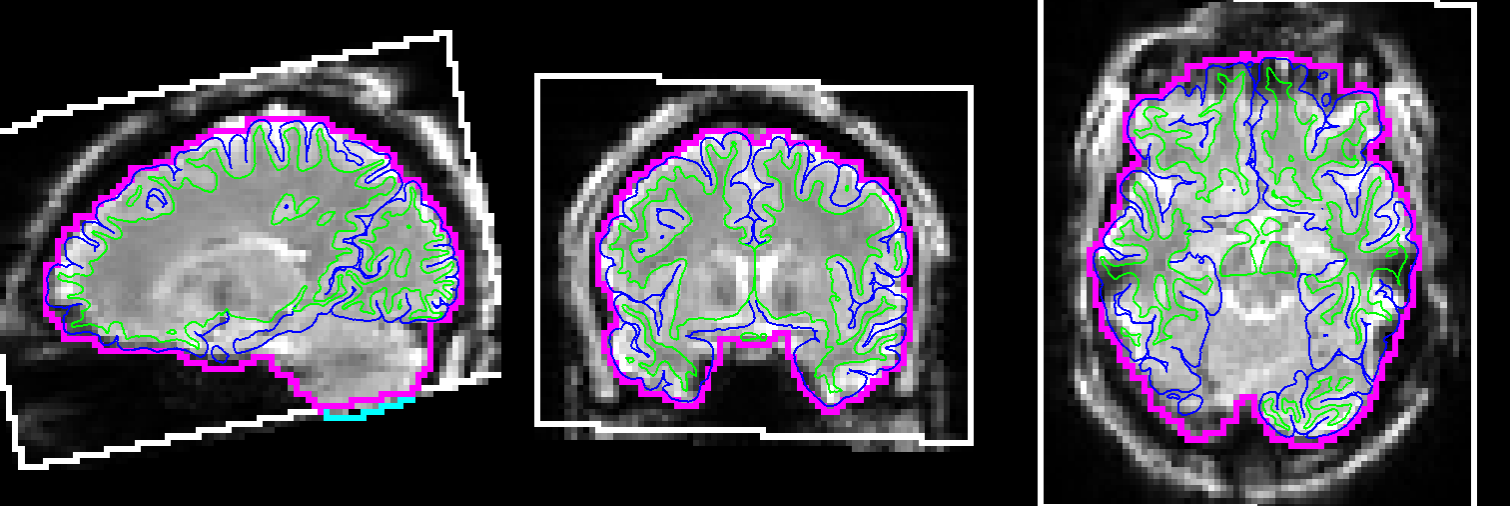
Workbench scene produced by the pipeline to assess registration and masking accuracy. The FreeSurfer white and pial surfaces are shown via thin green and blue lines, respectively. The ASL volumetric brain mask outline is shown in magenta. The white box denotes the field of view of the ASL acquisition, transformed into the ASL-gridded T1 w space. The cyan line (seen at bottom of cerebellum in the sagittal view) denotes a section of the ASL brain mask that lies outside the field of view. The base image in greyscale is the first volume of the fully corrected ASL timeseries image.

## 3 Results

### 3.1 Data pre-processing

**Fig. 5.**
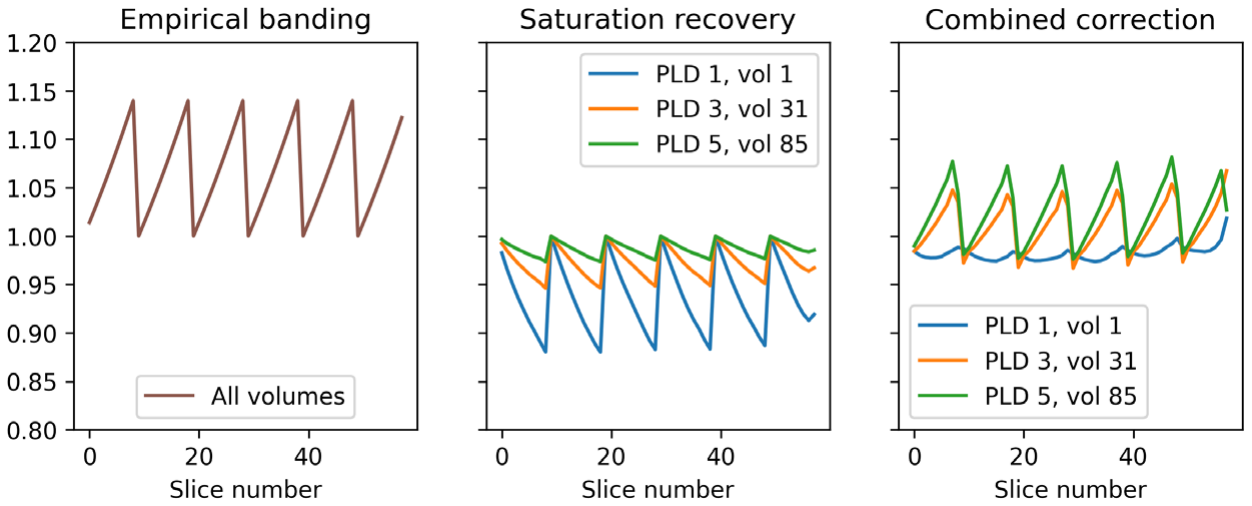
Magnitude of the two multiplicative banding corrections according to slice number and PLD/volume number within the ASL timeseries. First panel: the empirical banding correction applies equally to all PLDs/volumes within the ASL timeseries. Second panel: the saturation recovery correction is stronger for early PLDs than later ones (0.2 s vs. 1.2 s vs. 2.2 s, respectively) and operates in the opposite direction to the empirical banding correction. Final panel: when the two corrections are combined, they almost cancel each other in early PLDs and have a stronger effect in later PLDs (due to the reduced magnitude of the saturation recovery correction).

Figure 5shows the effect of the two individual banding corrections (empirically derived and saturation recovery) individually and combined together on a slice-wise basis. The two banding mechanisms worked in opposing directions: at early PLDs, the saturation recovery effect was almost equal and opposite to the empirical banding effect, leading to a subtle overall correction, whereas for late PLDs where the saturation recovery effect was weaker, the empirical banding effect dominated.

**Fig. 6.**
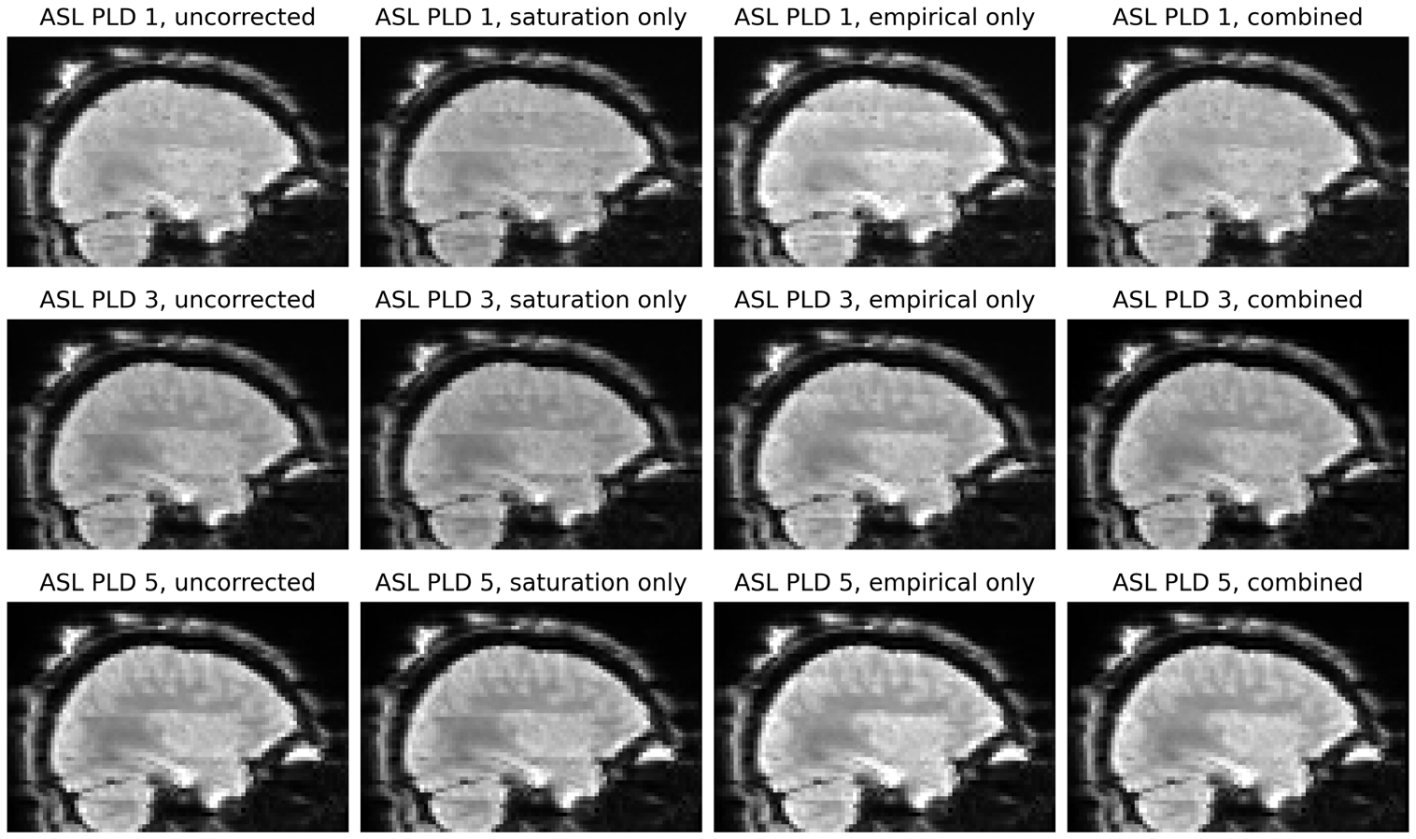
Banding artefacts visible in the first (0.2 s), third (1.2 s), and final (2.2 s) PLDs of the ASL timeseries. First column: before correction, visible banding varies according to PLD, because the saturation recovery mechanism affects each PLD differently. Second column: saturation recovery correction in isolation is unable to remove all banding at any PLD. Third column: the empirical banding correction in isolation does not remove banding in the early PLDs but does so in later PLDs. Fourth column: at all PLDs, the combination of the two banding corrections better removes banding than in isolation. The images in all columns are single volumes from the corresponding PLD group, and were previously corrected for gradient non-linearity distortion, susceptibility distortion, and the receive-coil bias field.

Figure 6shows the effect of the two banding corrections applied in isolation and together to the ASL timeseries from participant HCD0378150. The saturation recovery correction in isolation had minimal effect on the volumes of the ASL series with the longest (2.2 s) PLDs. This was consistent with theoretical prediction: at longer PLDs, the relative differences in slice-timing decrease, which will reduce the prominence of the saturation recovery effect, as was observed. Conversely, the empirical correction in isolation was unable to fully remove banding on the shortest (0.2 s) PLDs, for which the saturation recovery effect is substantial. For all PLDs, the combination of the two separate corrections was better able to remove banding than in isolation.

### 3.2 Group average CBF and ATT maps

**Fig. 7.**
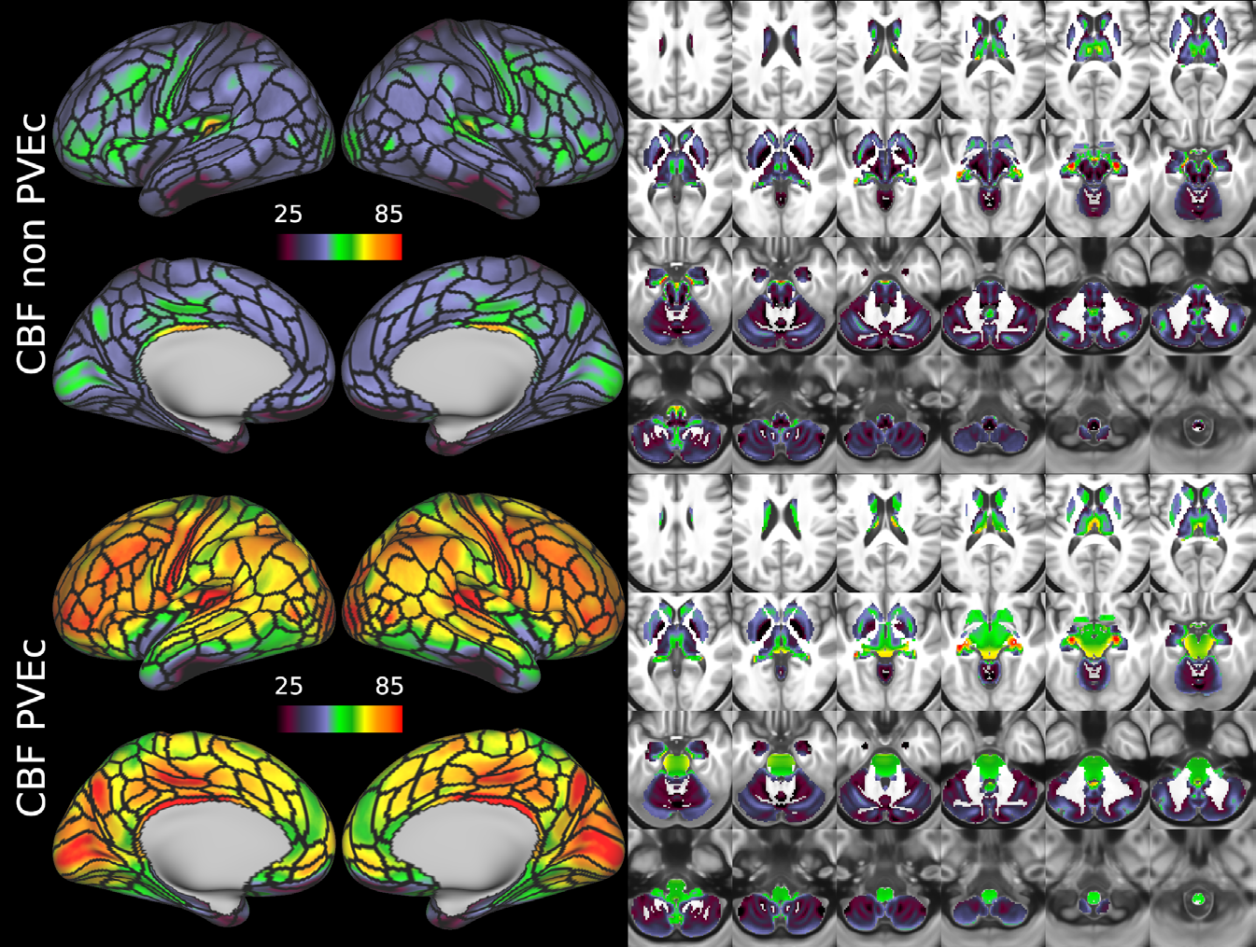
Group average CBF maps (in ml/100 g/min) for the HCA cohort in the cortex and subcortical structures. PVEc led to substantial increases in CBF in the cortical ribbon; the increase was less pronounced in the subcortex.

Figure 7shows the group average CBF maps for the HCA cohort, both with and without PVEc. In these and the following figures, the HCP multi-modal cortical parcellation (version 1.0) has been overlaid in black on the surface (Glasser, Coalson, et al., 2016). Areas of differential CBF (colour transitions) were observed to follow areal boundaries on the cortical surface. PVEc notably increased CBF, particularly in cortical tissue.

**Fig. 8.**
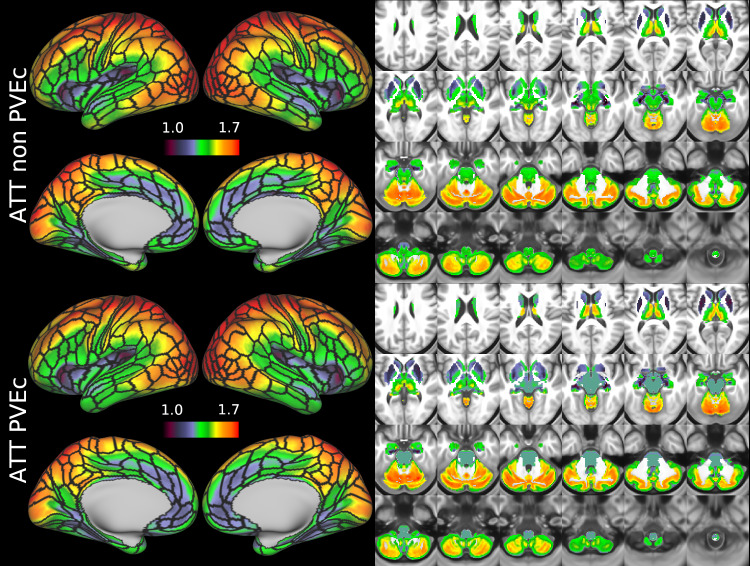
Group average ATT maps (in seconds) for the HCA cohort in the cortex and subcortical structures. PVEc did not lead to notable changes in ATT.

Figure 8shows the group average ATT maps for the HCA cohort, both with and without PVEc. Along with the volumetric representation given inFigure 11, ATT showed correspondence with known vascular anatomy, namely that the cores of the vascular territories (middle, anterior, and posterior cerebral arteries; MCA, ACA, PCA) were perfused before watershed regions that lie along the margins of these vascular territories; and anterior circulation (supplied by the MCA and ACA) was perfused before posterior circulation (supplied by the PCA). PVEc had a negligible effect on ATT.

**Fig. 9.**
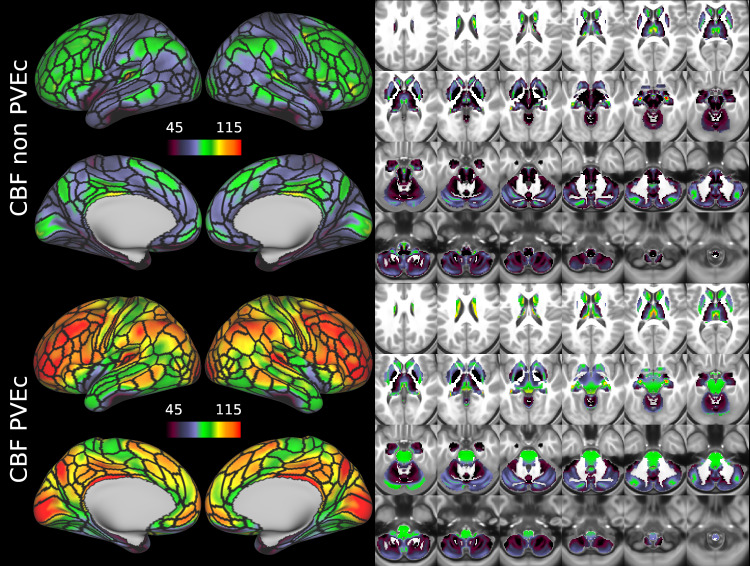
Group average CBF maps for the HCD cohort in the cortex and subcortical structures. PVEc led to substantial increases in CBF in the cortical ribbon; the increase was less pronounced in the subcortex.

Figures 9and10show group average CBF and ATT maps for the HCD cohort, both with and without PVEc. The ATT maps showed longer transit times for posterior and watershed regions.

**Fig. 10.**
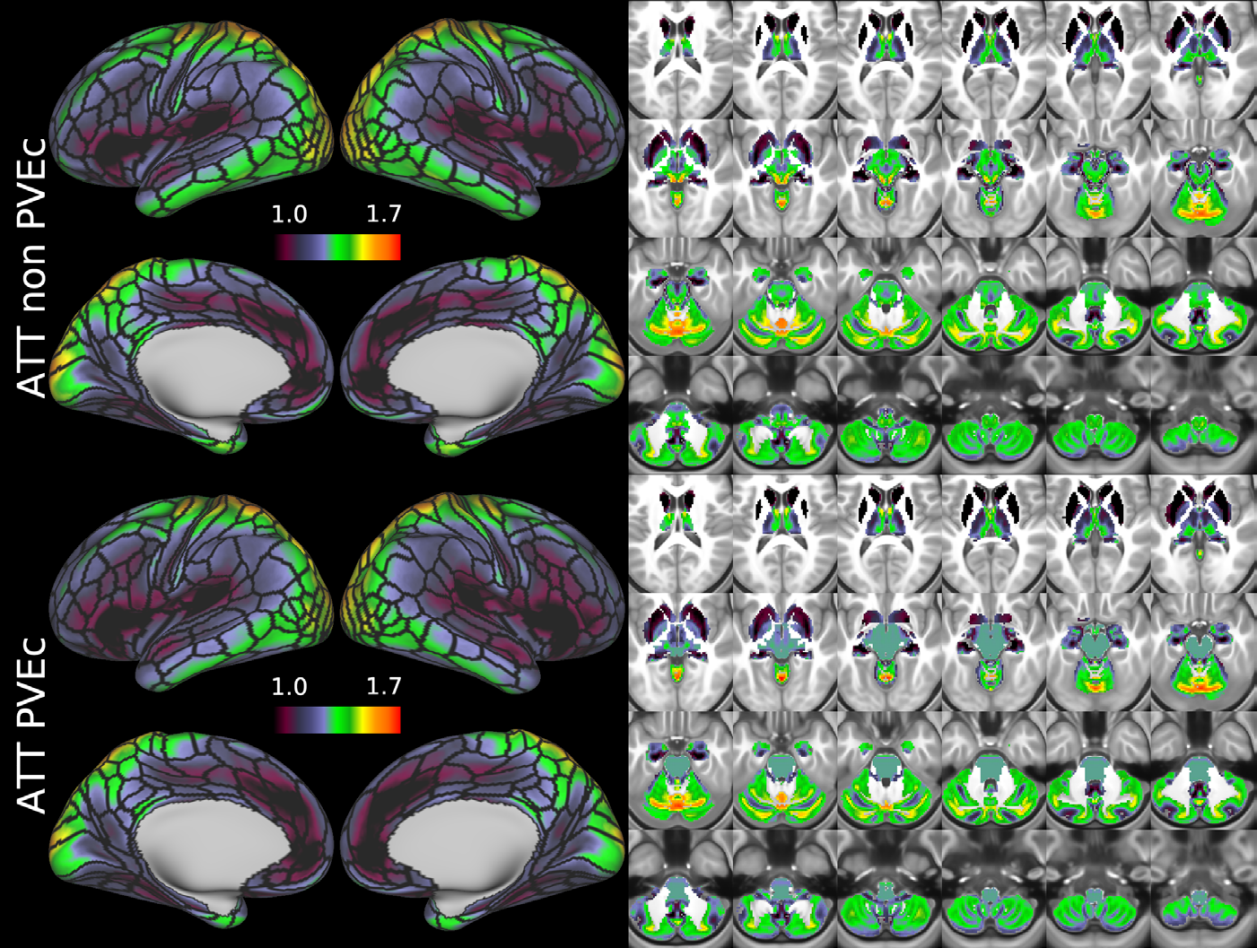
Group average ATT maps for the HCD cohort in the cortex and subcortical structures. PVEc did not lead to notable changes in ATT.

**Fig. 11.**
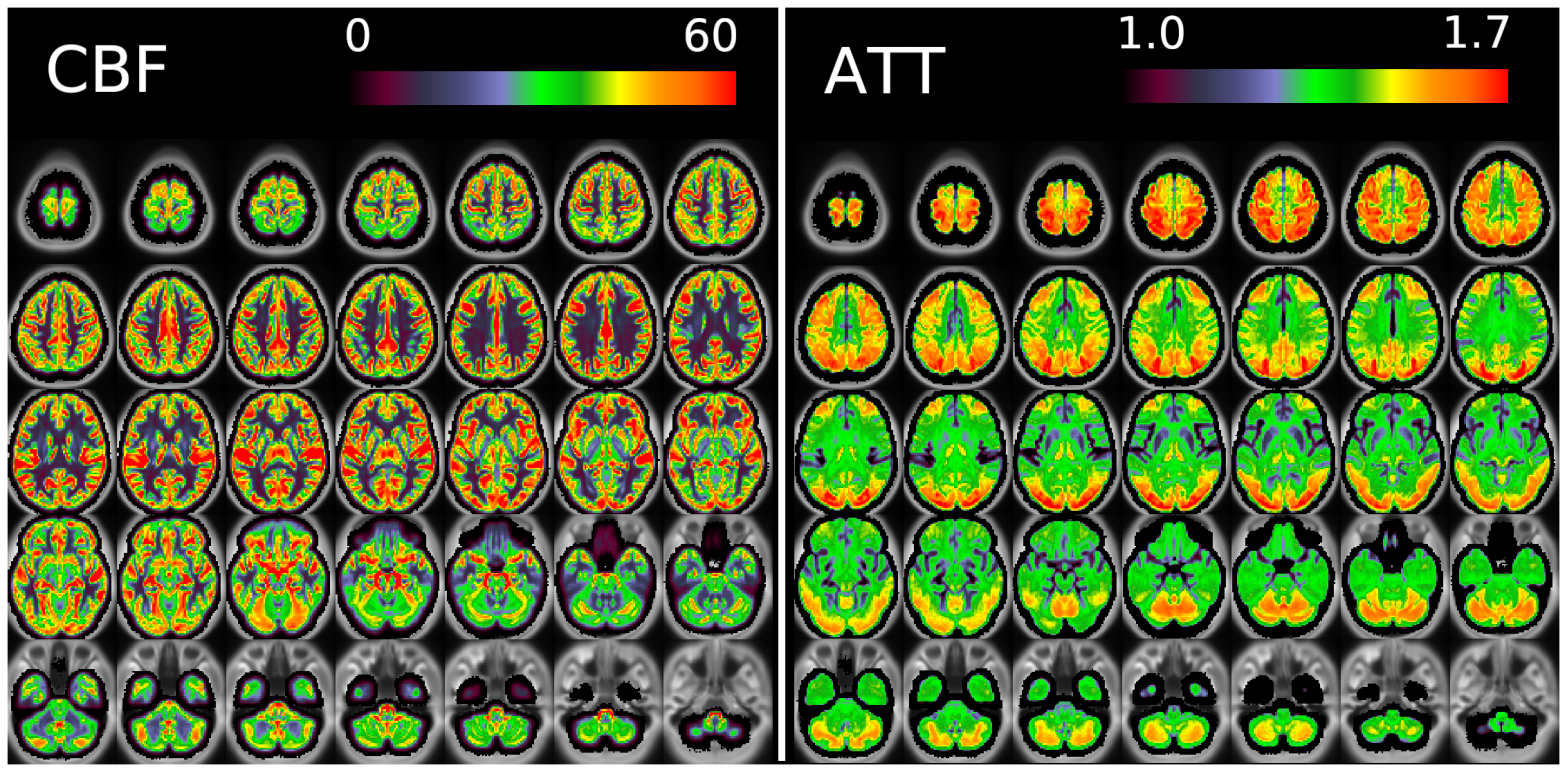
Group average CBF and ATT maps without PVEc for the HCA cohort in volumetric representation.

Figures 11and12show group average maps for non-PVEc CBF and ATT in each cohort, shown in a volumetric representation. This is a conventional representation of the same data shown in the preceding figures.

**Fig. 12.**
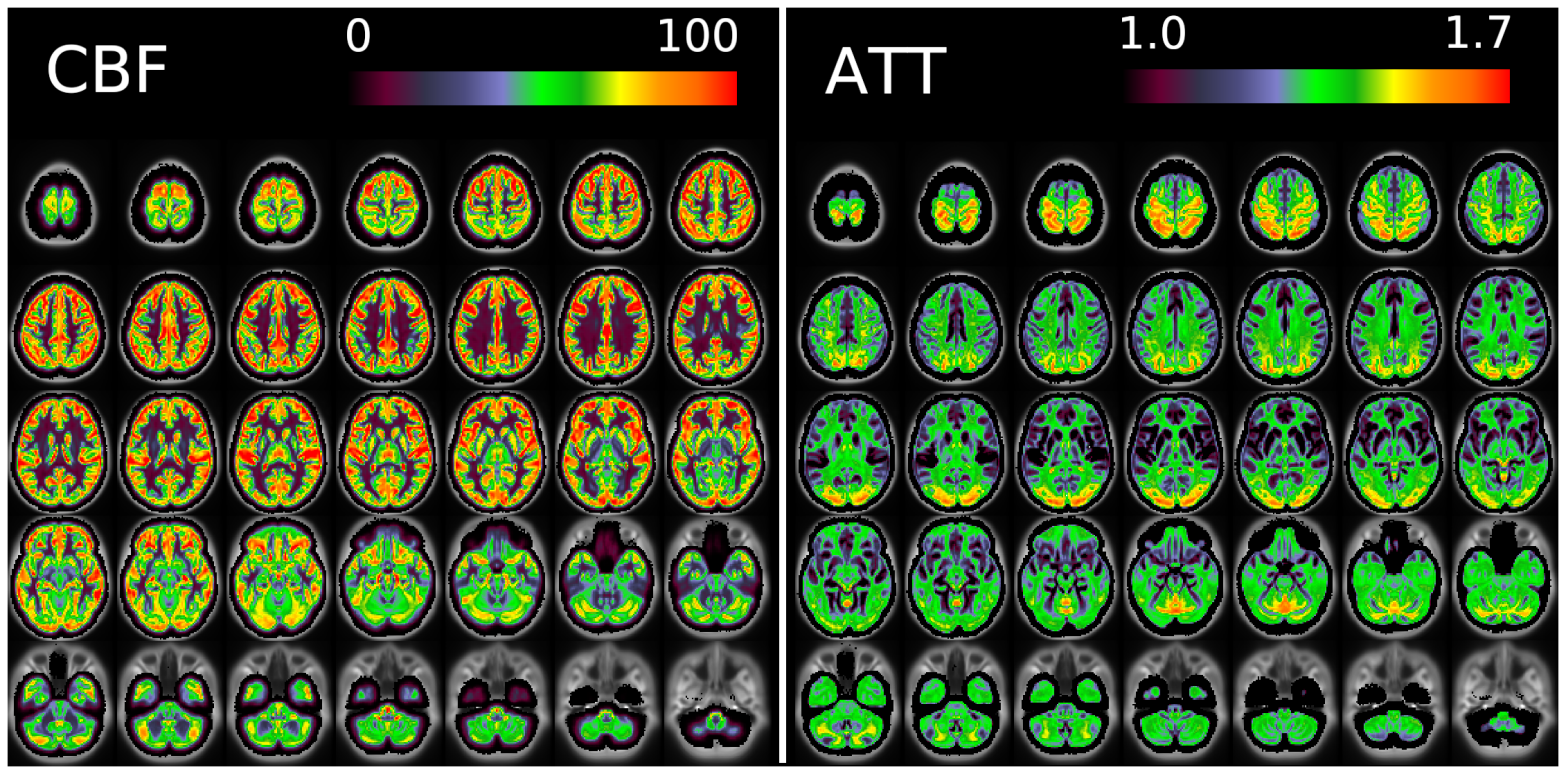
Group average CBF and ATT maps without PVEc for the HCD cohort in volumetric representation.

### 3.3 Summary statistics of perfusion and the effect of partial volume correction

**Fig. 13.**
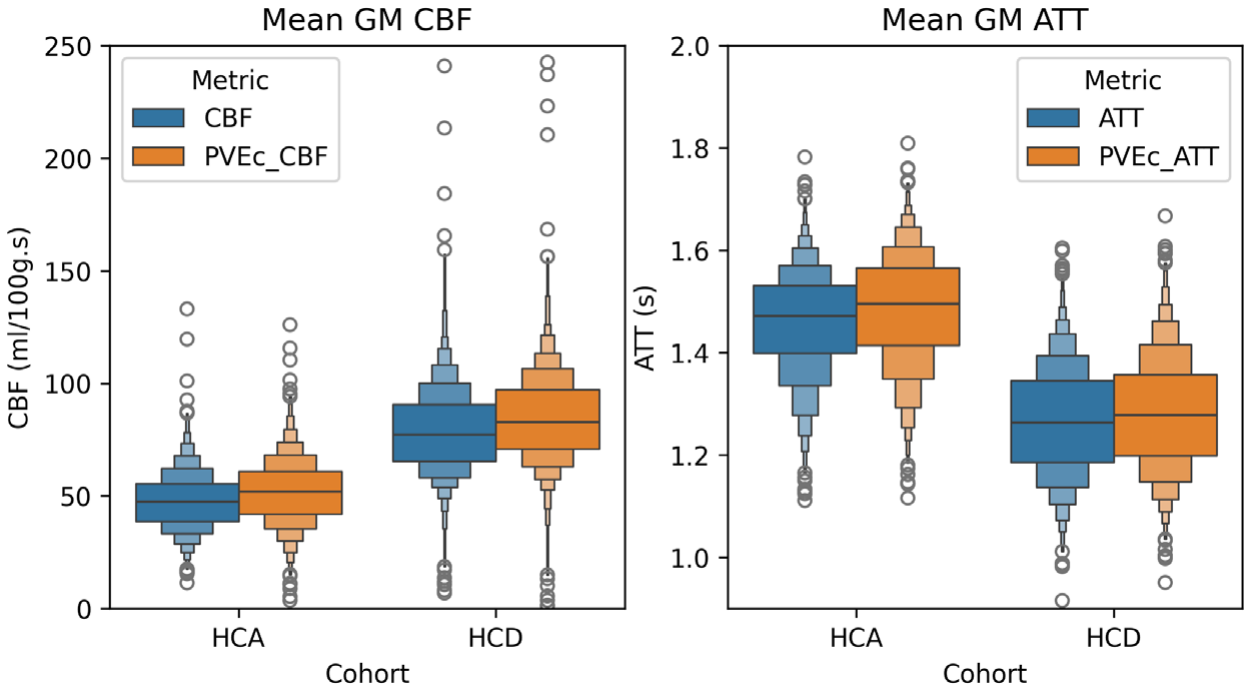
Mean GM CBF and ATT across individuals without and with PVEc for the two cohorts.

Figure 13shows distributions of each subject’s mean GM CBF and ATT before and after PVEc, grouped by cohort (the corresponding distributions for WM are given in theSupplementary Material).

Numerical values for each distribution mean are given inTable 1. Between cohorts, statistically significant differences were observed (all comparisons with*p*< 0.05). On the non-PVEc data, the HCA cohort had lower CBF than HCD (45 vs. 68 ml/100 g/min) and longer ATT (1.46 vs. 1.28 s).

**Table 1.** Mean GM parameter values within cohorts.

Parameter	HCA	HCD	Δ (HCD - HCA)	t-statistic	p-value	Cohen’s d
PVEc CBF	51.93	84.35	32.42	51.17	<1e-10	1.81
CBF	47.69	79.01	31.32	49.30	<1e-10	1.75
PVEc ATT	1.49	1.28	−0.21	−52.36	<1e-10	−1.84
ATT	1.46	1.27	−0.19	−51.83	<1e-10	−1.82

The differences between cohorts for both PVEc and non-PVEc values were statistically significant (independent t-test not assuming equal variance) in all cases. Cohen’s d was calculated for independent samples using pooled variance.

Table 2shows that PVEc significantly increased GM CBF in both cohorts: from 45 to 61 ml/100 g/min in HCA and from 68 to 85 ml/100 g/min in HCD. For both cohorts, PVEc led to a small but statistically significant decrease in GM ATT on the order of 0.01 s.

**Table 2.** Within cohorts, PVEc lead to large changes in CBF and small changes in ATT.

Cohort	Parameter	Non-PVEc	PVEc	Δ (PVEc – non-PVEc)	t-statistic	p-value	Cohen’s d
HCD	CBF	79.01	84.35	5.34	38.61	<1e-10	0.97
HCD	ATT	1.27	1.28	0.02	29.35	<1e-10	0.74
HCA	CBF	47.69	51.93	4.24	57.12	<1e-10	1.40
HCA	ATT	1.46	1.49	0.03	46.26	<1e-10	1.13

In all cases, the difference between PVEc and non-PVEc was statistically significant (paired t-test). Cohen’s d was calculated for paired samples as the mean of differences divided by the standard deviation of differences.

**Fig. 14.**
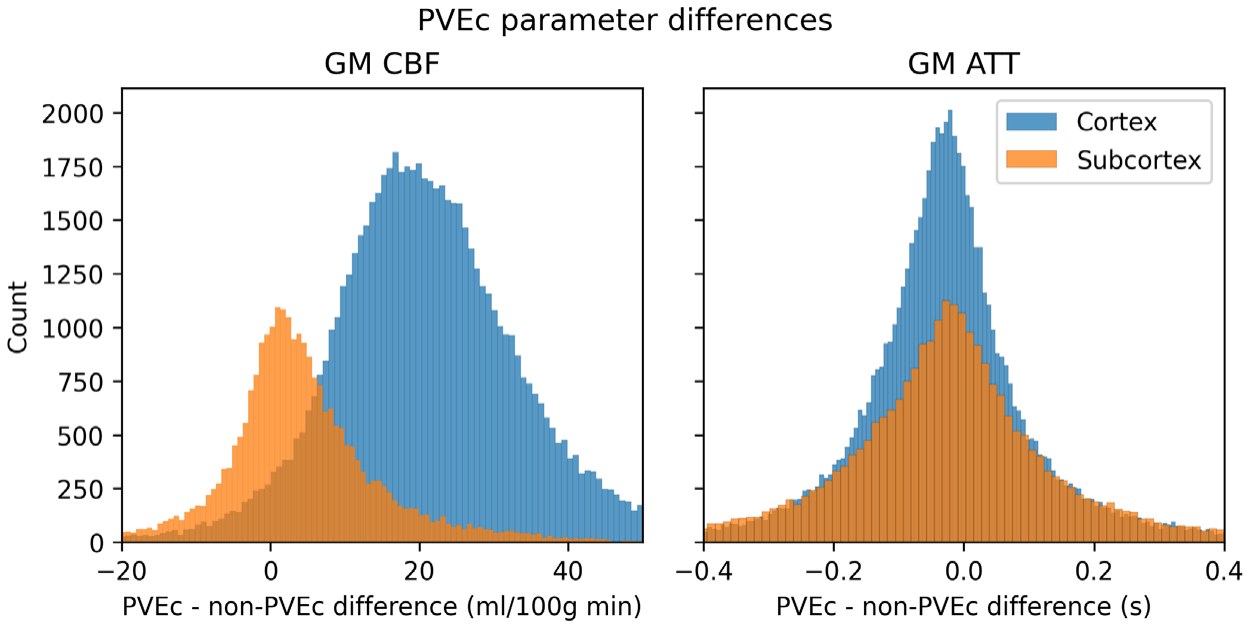
The effect of PVEc on CBF (left) and ATT (right), shown as a histogram of differences in cortical and subcortical greyordinates for a single HCD subject. In the cortex, the mean increase in CBF was around 15 ml/100 g/min, whereas in the subcortex, the increase was around 5 ml/100 g/min. For ATT, the mean difference was close to 0 s and there was no substantial difference in the distribution between cortical and subcortical greyordinates.

Figure 14shows the effect of PVEc on GM CBF and GM ATT measurements, separated into subcortical and cortical greyordinates, for a single subject. For cortical greyordinates, the average increase in CBF following PVEc was around 15 ml/100 g/min, whereas for subcortical greyordinates, it was around 5 ml/100 g/min. For ATT, both cortical and subcortical greyordinates showed negligible average increases.

### 3.4 Individual subject CBF and ATT maps

**Fig. 15.**
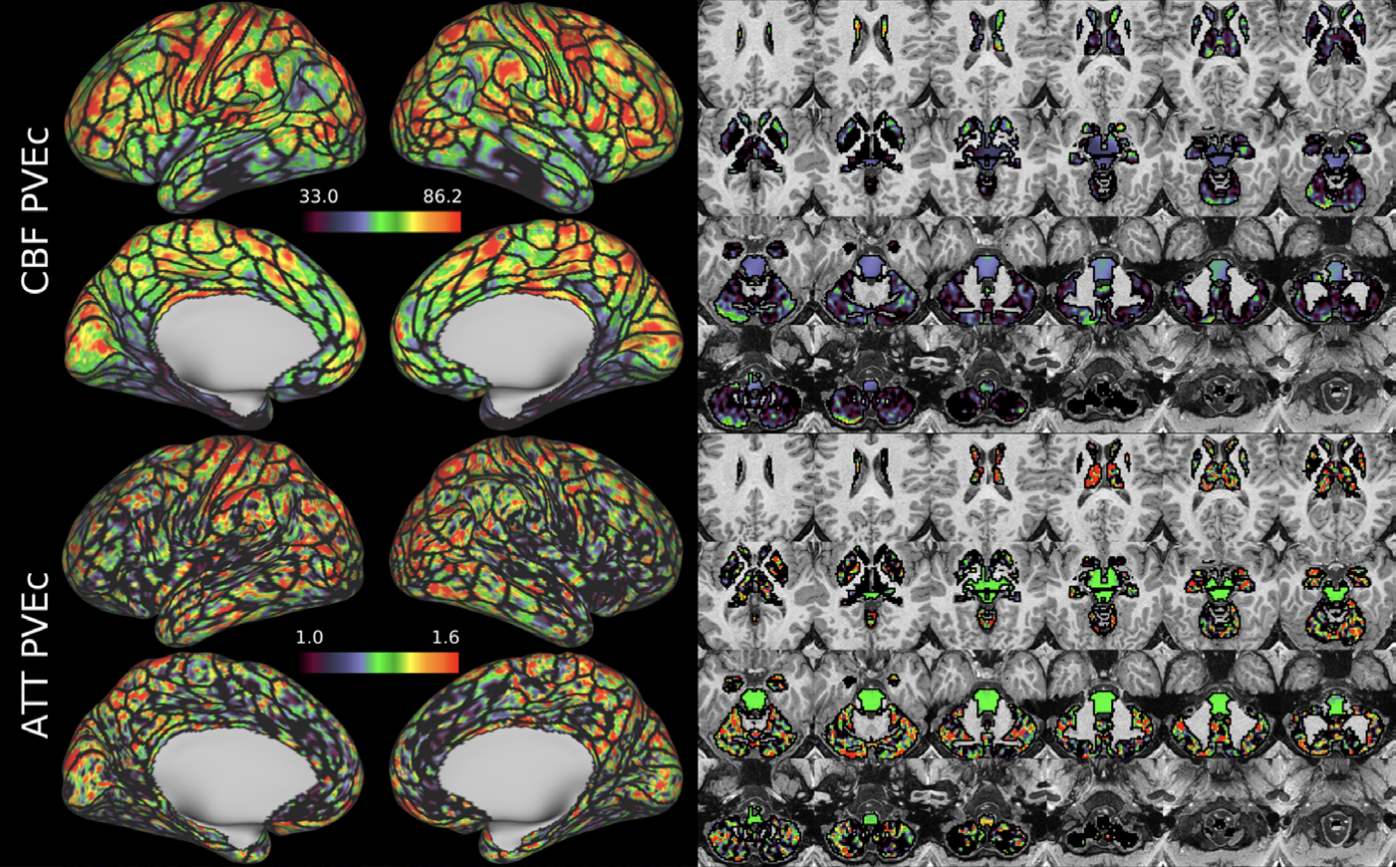
CBF and ATT maps (with PVEc) in the cortex and subcortical structures for subject HCD0378150 of the HCD cohort.

Figure 15shows CBF and ATT maps for a single participant of the HCD cohort. Although the individual subject maps were noisier than the group average, it was possible to observe similarities with the group average maps; for example, elongated ATT in posterior and superior regions of cortex.

**Fig. 16.**
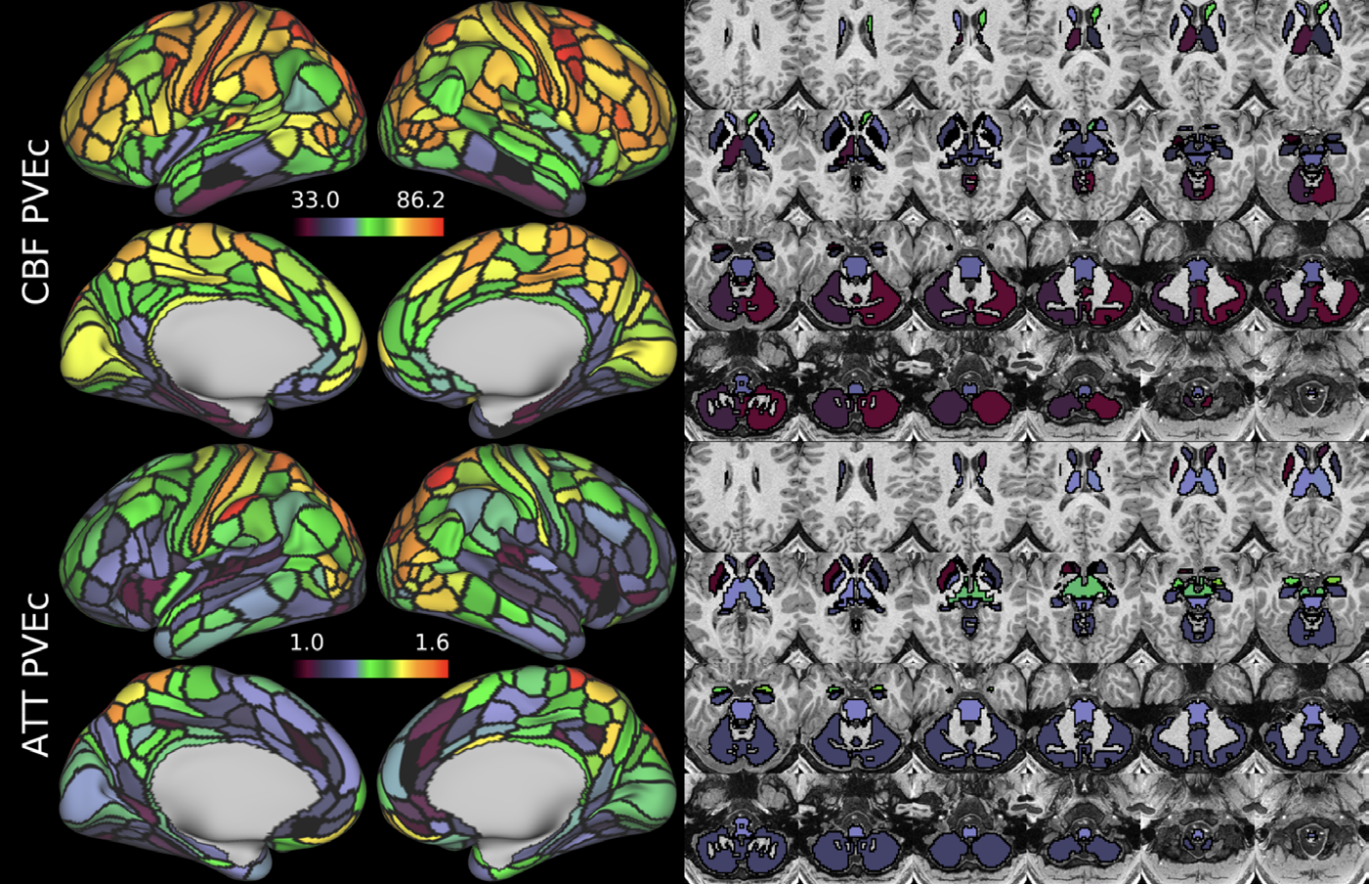
shows the same individual and measures asFigure 15represented as IDPs using the HCP multi-modal parcellation’s cortical areas and major subcortical structures.

Figure 16shows the same HCD individual asFigure 15, where CBF and ATT have been averaged within the parcels of the MCP multi-modal parcellation. This reduced the dimensionality of the data dramatically, enabling the data inFigure 12to be represented in 379 parcels (whereas there are 91,282 greyordinates inFig. 11).

**Fig. 17.**
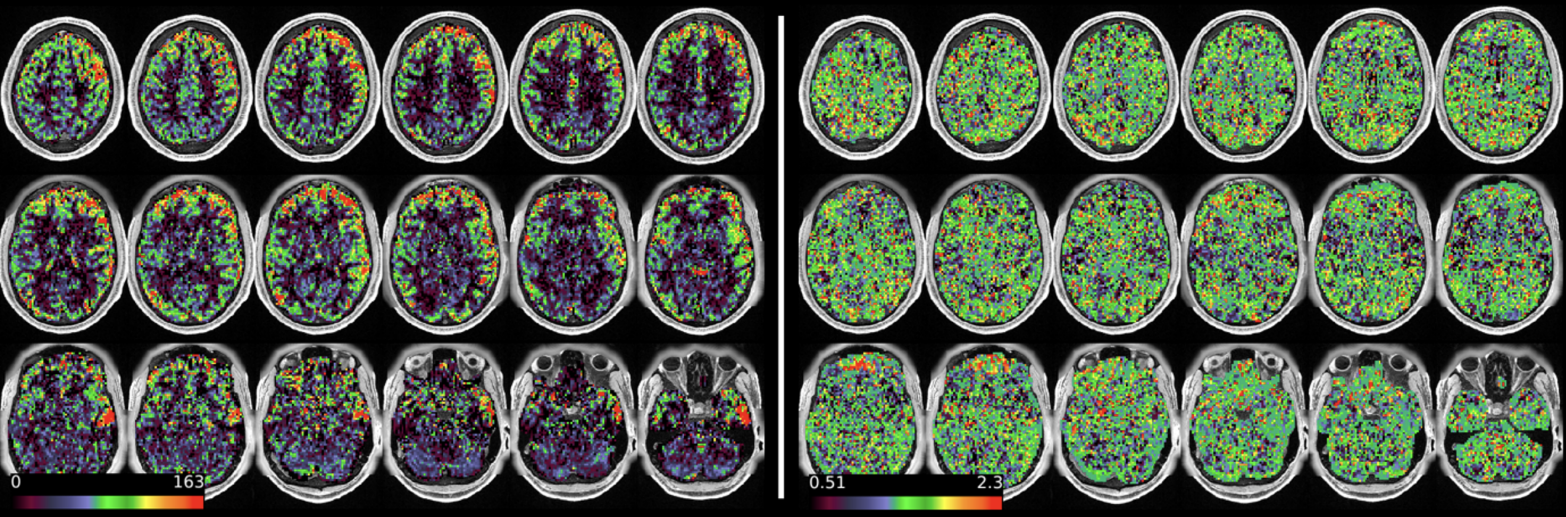
Volumetric CBF (left) and ATT (right, both non-PVEc) maps for subject HCD0378150 of the HCD cohort.

Figure 17shows volumetric CBF and ATT maps for a single subject of the HCD cohort. Though these appear to be noisy compared with typical ASL-derived maps, it should be noted they are at unusually high resolution and have not had any form of smoothing applied. Partial volume corrected variants of these maps are given in thesupplementary material.

## 4 Discussion

### 4.1 Haemodynamic measures: CBF and ATT

The HCP Lifespan ASL dataset and accompanying pipeline have yielded a dataset that is larger than existing ASL studies. The use of a high spatial resolution multiple delay acquisition has enabled CBF and ATT maps to be produced in both volumetric and surface representations. The group average surface-based ASL measures, which were generated using areal-feature-based surface registration with minimal smoothing, represent the most high-resolution maps of brain perfusion currently available, and will enable the wider exploration of surface-based analysis techniques for ASL. The utility of these surface maps has yet to be fully explored for ASL imaging, as it represents a significant departure for a modality that has nearly always been analysed in a volumetric manner.

CBF on the cortical surface was often observed to follow cortical areal boundaries (seeFigs. 7and9), which suggests that cerebral perfusion may be further regulated within vascular territories. Given that prior results from HCP fMRI data have established functional and/or connective relationships between cortical areas, it would be interesting to investigate whether perfusion is a further component of these relationships. Furthermore, given perfusion is a component of the BOLD effect, greater understanding of this parameter in the cortex will be of utility to fMRI studies. Comparing between cohorts, the HCD cohort had higher CBF than HCA (a difference of 24.7 ml/100 g/min), though the difference was slightly reduced by PVEc (23.4 ml/100 g/min), which is consistent with age-related atrophy leading to increased PVE presenting a confounding factor for perfusion measurement with ASL on elderly cohorts.

The ATT maps, both surface and volumetric, evidenced longer transit times in posterior portions than the anterior portions, which is expected because the vascular calibre of the common and internal carotid arteries that feed the ACA and MCA is larger than that of the vertebral arteries that feed PCA through the basilar artery, resulting in faster flow at a given blood pressure. Further, the HCA cohort showed higher GM ATT than HCD by ~0.2 s, which is consistent with previously reported findings that ATT increases with age (though the difference was smaller in WM, as detailed insupplementary material). The group mean volumetric ATT maps showed some small differences in GM/WM contrast between the two cohorts. One explanation for this could be reduced contrast in core/watershed ATT in the HCD cohort, making GM/WM contrast more prominent, whereas in the HCA cohort, core/watershed contrast dominates, possibly due to vascular disease.

Due to the high spatial resolution of the acquisition, the volumetric maps produced by the pipeline appeared noisier than ASL acquired at typical resolution (e.g., 3.4 x 3.4 x 5 mm). This was particularly the case in WM (shown, for example, inFig. 17). Not only does WM have intrinsically lower perfusion (and thus SNR) than GM, which poses a fundamental challenge for ASL, but also the acquisition itself was optimised for the cortex (high spatial resolution was used to facilitate accurate registration to the cortical surface, and the sensitivity profile for the multi-channel coil used preferentially increased SNR in the cortex). Under the Bayesian framework used by the FSL BASIL toolbox for perfusion quantification, parameter values will revert to their prior mean values when the data are uninformative. For WM ATT voxels, this is a likely explanation for why values tend to be near the prior mean of 1.6 s, although this would be expected to only have a small effect on the estimated perfusion value. Ultimately the pipeline presented here has not been optimised for WM voxels and more precise results might be achieved by averaging over regions in WM.

Nevertheless, recent work investigating high-resolution ASL has found it to be practical and feasible for perfusion measurement in the cortex and subcortex, despite the appearance of noise, because the reduction of inherent partial volume effects improves the localisation of perfusion without impacting sensitivity (Kashyap et al., 2024). A conventional mitigation for noise would be to perform spatial smoothing (similar to reducing spatial resolution), at the loss of spatial precision. The alternative strategy adopted here is to average perfusion measurements within neuroanatomically well-defined subdivisions to make efficient use of available SNR. This should increase statistical sensitivity for effects, while reducing dimensionality (thereby reducing the penalty for correction of multiple comparisons) without the need for indiscriminate spatial smoothing. These imaging-derived phenotypes should facilitate novel statistical analyses such as relating CBF and ATT to behaviour, cognition, genetics, or clinical conditions.

In both cohorts, PVEc was observed to lead to statistically significant increases in CBF estimates. This is despite the relatively high spatial resolution of the ASL data (2.5 mm isotropic), which reduces but does not eliminate the presence of PVE in the data. Indeed,Figure 14shows that PVEc led to greater increases in cortical than subcortical CBF, which is consistent with there being more PVE present in cortical voxels. Recent work has noted the fundamental trade-off inherent to PVEc, while acknowledging that both PVEc and non-PVEc data have value depending on the research question (Chappell et al., 2021). Without PVEc, differences in GM PVE between individuals due to differences in anatomy are encoded into the perfusion values, thus becoming a source of between-individual variability. Though PVEc mitigates this, it also increases the complexity of the model by introducing tissue-specific parameters which could result in over-fitting, introducing a different source of between-individual variability.

The brainstem was observed to have relatively high CBF after PVEc. This could be due to inaccuracy of PV estimates when segmenting this region of the brain (which would directly impact PVEc), the presence of a stronger bias field in the middle of the brain, or macrovascular contamination from nearby arteries. At any rate, it is important to note that perfusion measurement via ASL in WM is inherently difficult due to the low SNR associated with this tissue.

### 4.2 Correction of acquisition artefacts

The novel nature of the multi-band acquisition and the high demands for precision in spatial localisation in the HCP-Style approach to data acquisition and analysis (Glasser, Smith, et al., 2016) necessitated careful pre-processing of the ASL and calibration data to bring it into T1w alignment space at the original voxel resolution with properly normalised image intensities. The pipeline that has been implemented represents a best attempt at preprocessing given the unique nature of the acquisition, though it has not been compared against existing ASL pipelines. Recognising that users may wish to implement their own alternative kinetic modelling to the variational Bayesian method used in this work, the HCP-ASL processing pipeline also provides fully corrected ASL and calibration volumetric data, which means that a bespoke perfusion estimation may be performed without needing to re-create the prior correction steps.

Addressing the SMS banding artefact was a particular pre-processing challenge. The banding remaining after correction for saturation recovery is suspected to be caused by magnetisation transfer effects, namely that the acquisition of one slice has spill-over effects on the adjacent slice acquired next. In the derivation of the empirical correction used in this work, it was found that the coefficients differed slightly between samples of the HCA and HCD cohorts (illustrated in theSupplementary Material). This could be due to age-related differences in grey matter and white matter volumes. Ultimately it was decided to use a single set of correction coefficients derived over an equal-sized sample of both cohorts for simplicity, but in the future, a personalised correction technique to remove the need for a representative population might be feasible and would allow the pipeline to translate to unseen datasets with ease. A personalised approach could also be beneficial for application to subjects where tissue T1 may be modified by pathology, as this mechanism at least partially explains the banding artefact.

Across both cohorts, lower CBF was observed in the most inferior cortical regions such as the inferior temporal cortex and orbito-frontal cortex. It is likely that this was caused by uncorrectable susceptibility-induced signal loss. Though distortion correction can relocate signal to the correct position within the brain, it cannot correct the underlying loss of signal, which could only be solved by the use of a spin-echo acquisition.

### 4.3 Further work

An important limitation of the pipeline in its current form is that it does not implement any form of automated QC (instead providing some visual outputs that can be used as part of manual QC). In the context of the HCP ASL dataset, this means researchers must make their own judgement on which subjects to exclude from analysis. As for application of the pipeline to more heterogeneous or possibly pathological clinical data, this cannot be assumed to work without modification (particularly as some of the corrective steps implemented are acquisition specific). QC of motion estimation and correction would be a particularly important consideration for application of the pipeline to very young or elderly clinical subjects for which substantial motion is to be expected.

An area in which the pipeline could be improved is image denoising, potentially using independent component analysis (Carone et al., 2019). Preliminary work with this approach was unable to reliably identify noise components, so it was not adopted for the final pipeline. This could be due to the relatively limited number of timepoints in ASL data relative to fMRI, which reduces the performance of ICA (i.e., ICA’s performance is markedly improved in high spatial and temporal resolution fMRI relative to legacy low spatial and temporal resolution fMRI).

A further area of future development would be to adopt a fully surface-based approach to perfusion estimation. Since the BASIL toolbox operates in volume space, currently the pipeline produces all perfusion estimates in voxel space and then projects these onto the cortical surface. A more direct, and potentially more advantageous, approach would directly estimate perfusion on the cortical surface after projecting the ASL timeseries from volumetric data (Kirk, 2021). This would allow spatial regularisation (which is beneficial given the inherently low SNR of ASL data) to be applied in a manner that respects the existing HCP principle of constrained smoothing within neuroanatomically well-defined tissues or brain areas. Indeed, one could in theory directly model the ASL parameters within brain areas, a technique that has previously been shown to be beneficial for task-based fMRI GLM modelling (Glasser, Coalson, et al., 2016).

## 5 Conclusion

The HCP Lifespan ASL dataset is a large and high-quality resource that will enable perfusion to be studied in unprecedented detail during the development and ageing phases of life. The HCP-ASL pipeline has been developed as a best attempt to implement an HCP-Style data analysis while accounting for the specific nature of the acquisition with state-of-the-art correction techniques. Perfusion estimation has been performed on the pre-processed data using an established variational Bayesian method, though investigators can run their own perfusion estimation on the pre-processed data if they wish. Given ongoing debate on the utility of partial volume correction, particularly where ageing and pathology are concerned, both corrected and non-corrected perfusion estimates have been produced to provide investigators maximum flexibility. A preliminary group analysis revealed expected differences in haemodynamics between the development and ageing cohorts, specifically, reduced CBF and elongated ATT with increasing age.

## Supplementary Material

Supplementary Material

## Data Availability

The pipeline code is found athttps://github.com/physimals/hcp-asl/releases/tag/v0.1.2and the pre-processed data will be available at the NIH data archive (NDA):https://nda.nih.gov
