# Supplementary material for: Arterial spin labelling perfusion MRI analysis for the Human Connectome Project Lifespan Ageing and Development studies
Source: Imaging Neurosci (Camb). 2025 Jan 27;3:imag_a_00444. doi: 10.1162/imag_a_00444 (PMC11905292; doi:10.1162/imag_a_00444)
Supplement: Supplementary Material [file imag_a_00444-supp.pdf]

Arterial spin labelling perfusion MRI analysis for the Human Connectome Project Lifespan Ageing and Development studies: Supplementary Material

Data acquisition sites

The distribution of data acquisition across sites is given in the below table.

	Harvard University	Massachusetts General Hospital	University of California, Los Angeles	University of Minnesota	Washington University, St Louis
HCD	352	0	344	318	292
HCA	0	293	298	307	301

Supplementary Table 1: number of subjects acquired at each site

Saturation recovery T1 estimation

Supplementary Figure 1 shows an example T1 map estimated by FSL FABBER used to perform saturation recovery correction. The contrast in GM / WM T1 values means that these tissues will have been differentially affected by the saturation recovery effect, and thus require different amounts of correction which is achieved by using a voxel-wise map of T1 estimates.

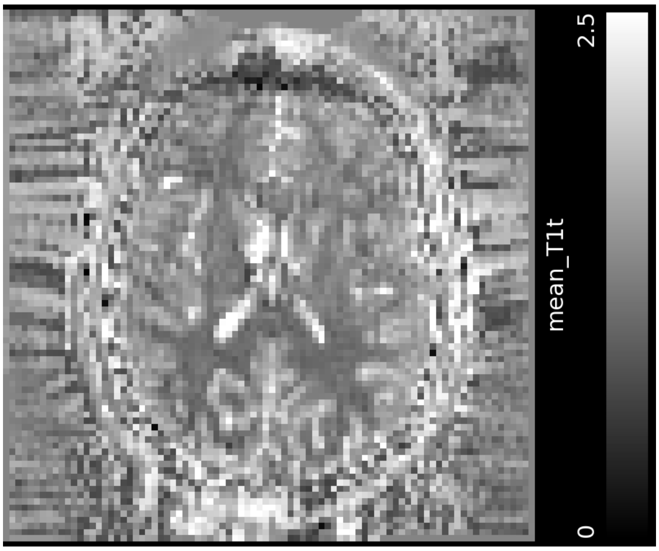

Supplementary Figure 1: Example T1 map (axial slice) generated by FABBER for a single subject, used for saturation recovery banding correction (units in seconds).

Derivation of the empirical banding correction

Saturation recovery correction alone was found to be insufficient to fully remove the banding artefact from the ASL and calibration data. The remaining banding is thought to arise from magnetisation transfer effects, for which separate empirical correction factors were derived in the following manner using 40 randomly selected participants from each of the Aging and Development cohorts.

First the M0 images were corrected for saturation recovery and bias field effects as described in the Methods. Grey matter (GM) and white matter (WM) FreeSurfer segmentation masks were taken from the structural pre-processed outputs of each participant's T1w anatomical image and linearly registered with the corresponding M0 images using the registrations derived earlier in the pipeline. In the space of the M0 images, the GM and WM masks were unified to produce a brain tissue mask, which was used to calculate the mean signal intensity within each slice of the M0 image. The slice-wise mean signal intensities were averaged across the subset of each cohort to generate a slice-wise population-averaged mean M0 signal intensity profile. The profiles for each cohort (given in Supplementary Figure 2) showed an approximately linear decrease in M0 signal intensity over the 10 slices in each of the central four slice stacks. The magnitude of this effect relative to the signal intensity of the first slice in each band was estimated by fitting a single linear function to the mean of the central four bands. Correction was then performed by multiplying the image data in each band by the inverse of the linear function. The signal intensities in the two outermost (inferior and superior) bands were not used to calculate the banding correction due to the low number of tissue voxels in these bands leading to high variability, though the fit derived from the central four bands was extrapolated to cover all six bands when performing the correction. Finally, the mean of the coefficients derived in each cohort was taken to give a single overall set of corrective coefficients. Supplementary Figure 3 gives an schematic of this process.

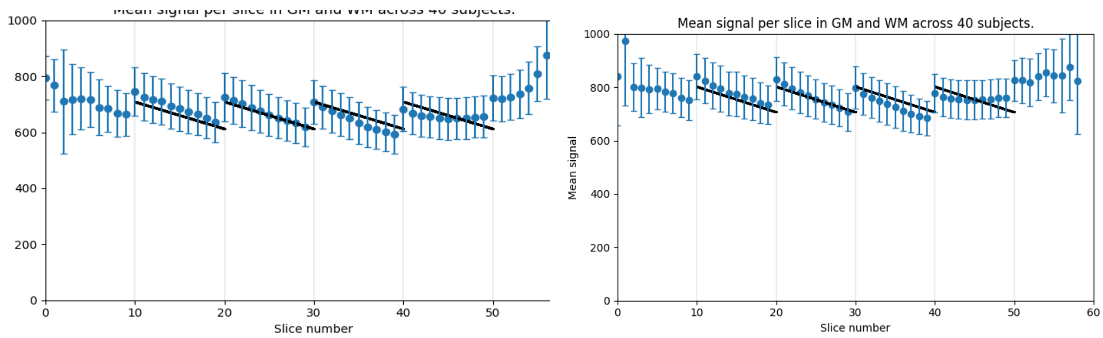

Supplementary Figure 2: Mean calibration image signal in WM and GM by slice number for 40 HCA and HCD subjects.

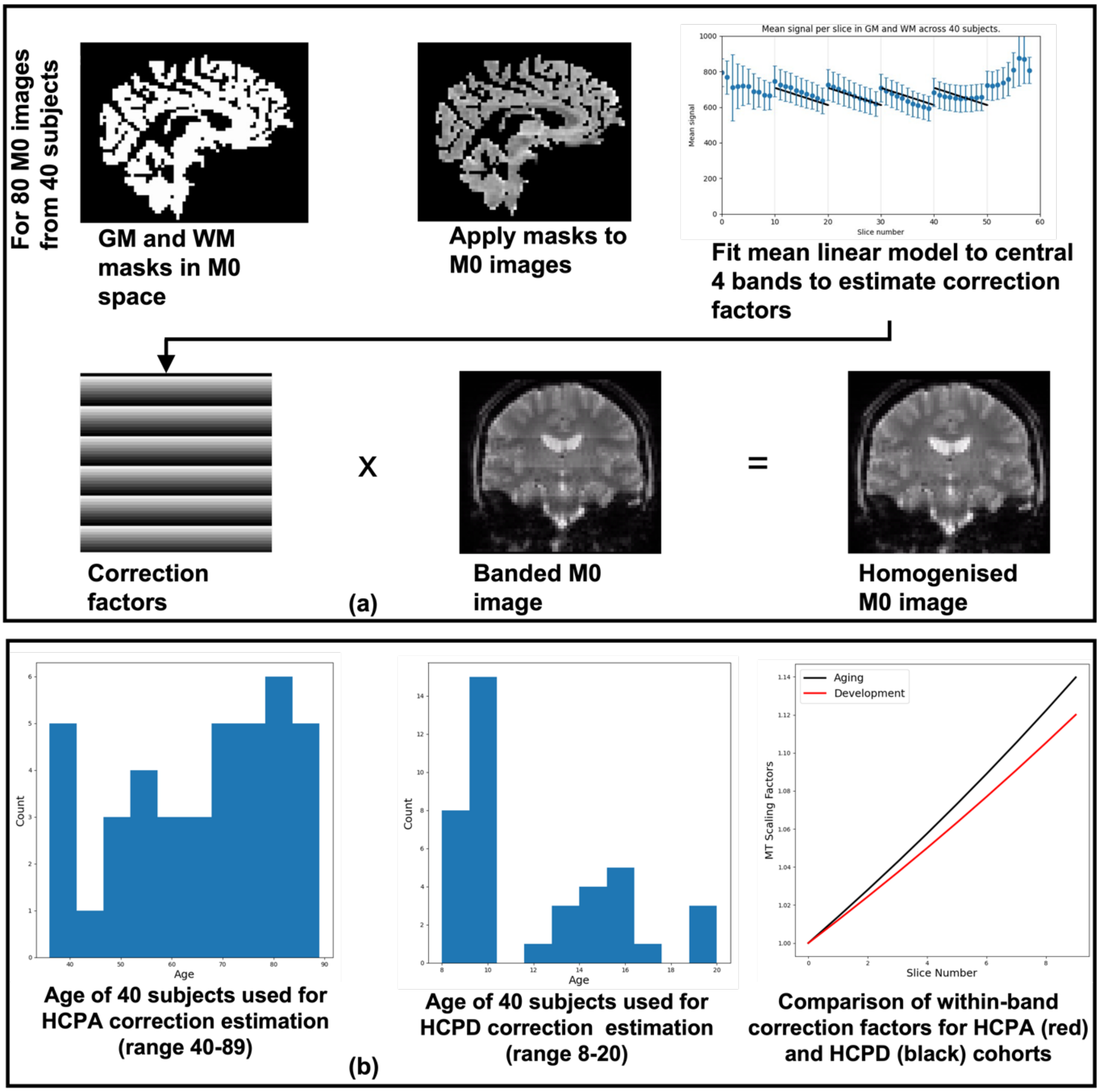

Supplementary Figure 3: Process used to produce population derived scaling factors to correct for slice-wise intensity variations (a), thought to arise from an MT effect resulting from the use of a SMS acquisition to achieve high resolution for the HCP Lifespan ASL data. (b) shows the age distributions of the 40 subjects used to derive correcting factors for the Ageing (left) and Development (centre) cohorts. A comparison of the two sets of scaling factors is shown on the right of (b).

Comparison of standard and boundary-based registration (BBR)

Supplementary Figure 4 shows registration between the calibration image and cortical surfaces (in T1w alignment). The initial registration is performed directly between the calibration and T1w image; for the final registration, BBR has been used between the ASL space CBF map and T1w image to refine the registration, which is then combined with the earlier motion correction matrices to obtain a new calibration/T1w registration.

The difference between the two registrations is small, but slightly better for the final (refined) registration using the CBF map.

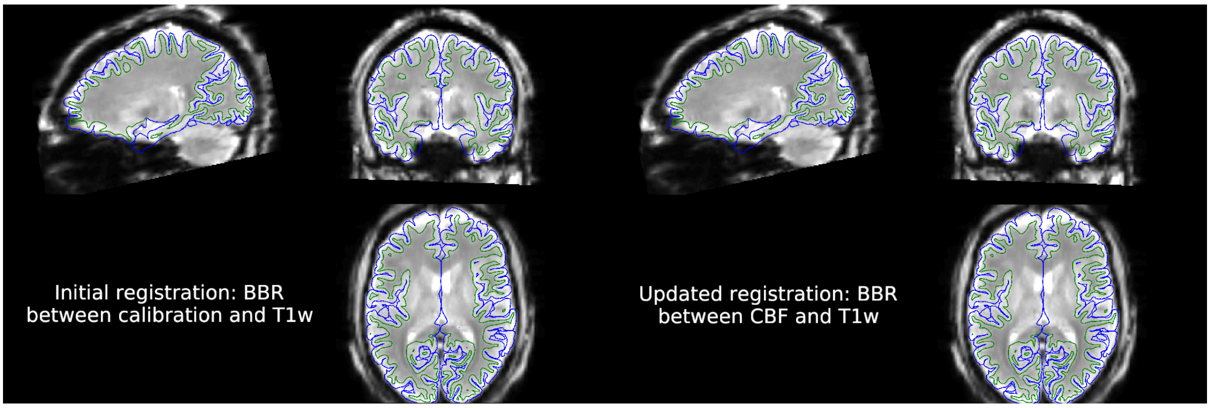

Supplementary Figure 4: Comparison of registration initially (left) and after final (right) registrations. The calibration image is shown in alignment with the cortical surfaces in T1w space. The initial registration is obtained using BBR between the calibration and T1w; at the second step, BBR between the CBF map and T1w has been combined with motion correction between the ASL and calibration image to yield an updated calibration/T1w registration. The difference in the translation between the two registrations is less than 1mm in each dimension.

Summary statistics of white matter perfusion

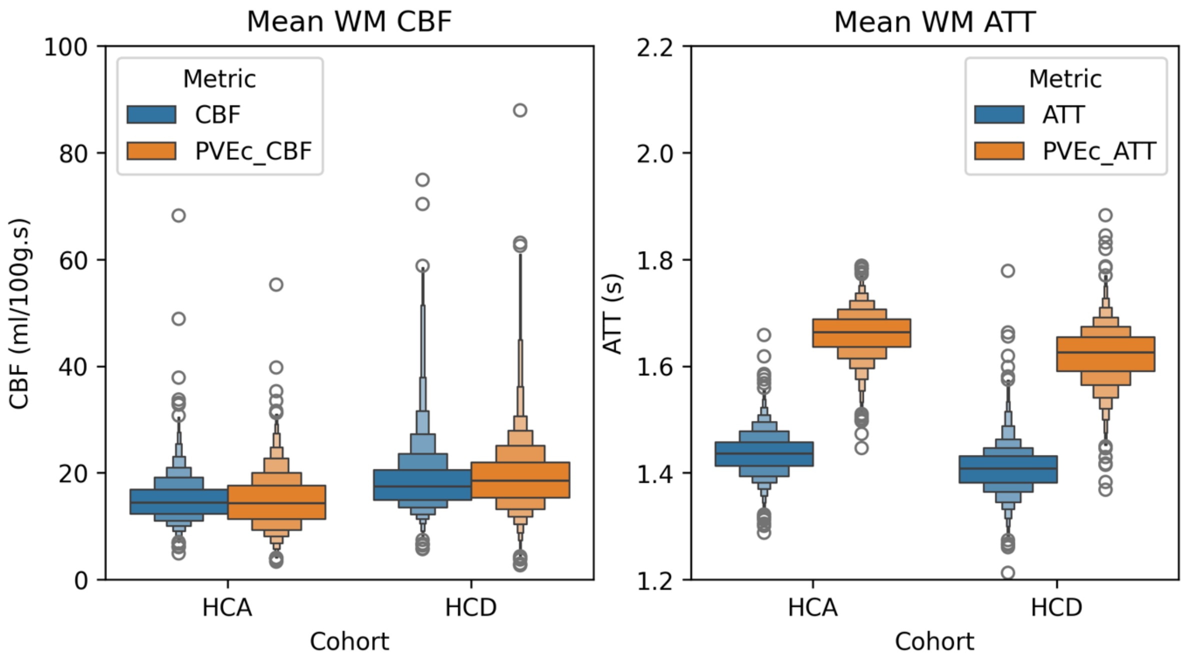

Supplementary Figure 5: Mean WM CBF and ATT across individuals without and with PVEc for the two cohorts. WM CBF was slightly higher, and ATT slightly lower, with and without PVEc in the HCD cohort. PVEc was observed to lead to a substantial increase in ATT for both cohorts; it should be noted that a longer prior was used for WM ATT (1.6s) during PVEc.

Parameter	HCA	HCD	Δ (HCD - HCA)	t-statistic	p-value	Cohen's d
PVEc CBF	14.72	19.63	4.91	16.25	< 1e-10	0.58
CBF	14.95	18.94	3.98	12.94	< 1e-10	0.46
PVEc ATT	1.66	1.62	-0.04	-23.74	< 1e-10	-0.84
ATT	1.44	1.41	-0.03	-20.19	< 1e-10	-0.71

Supplementary Table 2 Mean WM parameter values within cohorts. The differences between cohorts for both PVEc and non-PVEc values were statistically significant (independent t-test not assuming equal variance) in all cases. Cohen's d was calculated for independent samples using pooled variance.

Cohort	Parameter	Non-PVEc	PVEc	Δ (PVEc - nonPVEc)	t-statistic	p-value	Cohen's d
HCD	CBF	18.94	19.63	0.70	10.44	< 1e-10	0.26
HCD	ATT	1.41	1.62	0.21	318.85	< 1e-10	8.01
HCA	CBF	14.95	14.72	-0.23	-5.18	2.54e-07	-0.13
HCA	ATT	1.44	1.66	0.23	374.96	< 1e-10	9.19

Supplementary Table 3 Within cohorts, PVEc led to statistically significant changes in WM CBF and ATT (paired t-test). Cohen's d was calculated for paired samples as the mean of differences divided by the standard deviation of differences; the effect size of the change was small in CBF and larger in ATT.

Individual subject volumetric PVEc parameter maps

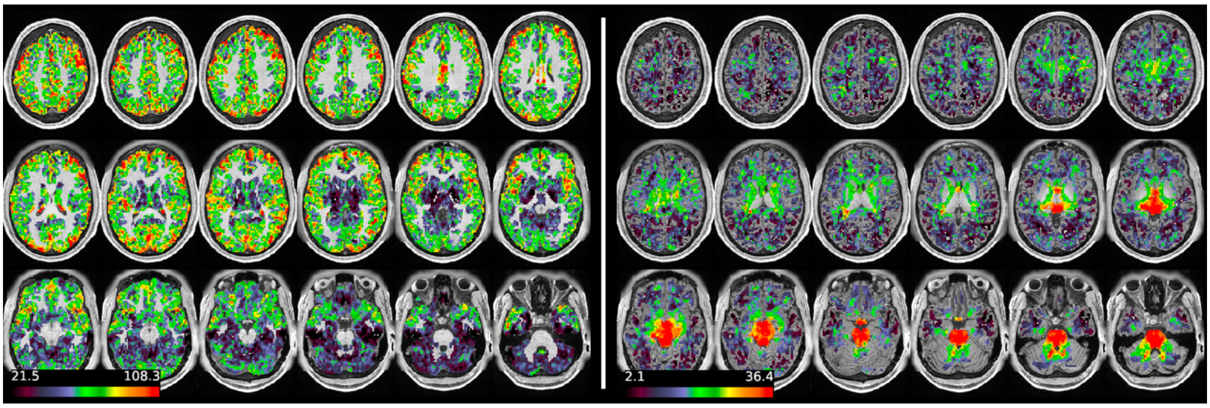

Supplementary Figure 6: Partial volume corrected CBF maps in GM (left) and WM (right), masked for voxels with >10% of each tissue, for subject HCD0378150 (chosen at random, and also presented in the main text).

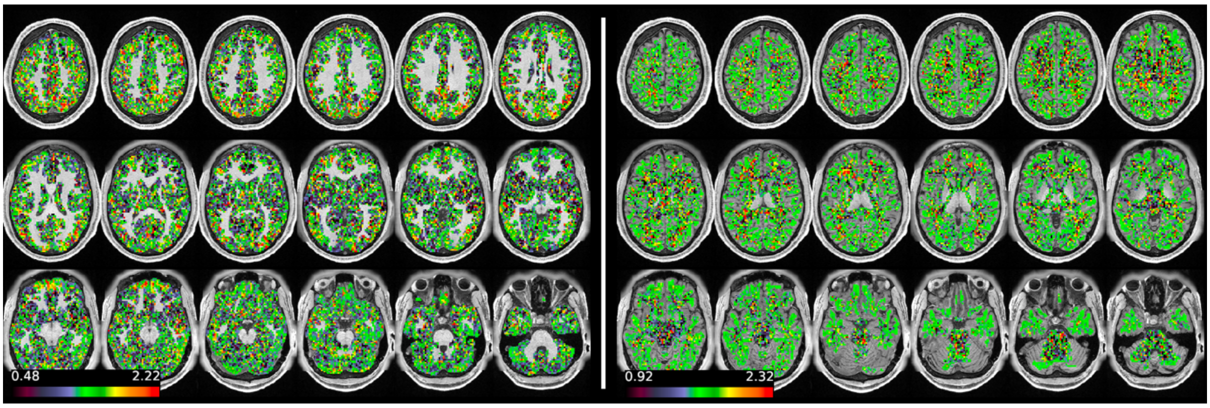

Supplementary Figure 7: Partial volume corrected ATT maps in GM (left) and WM (right), masked for voxels with >10% of each tissue, for subject HCD0378150 (chosen at random, and also presented in the main text).
